# NDUFAB1 confers cardio-protection by enhancing mitochondrial bioenergetics through coordination of respiratory complex and supercomplex assembly

**DOI:** 10.1038/s41422-019-0208-x

**Published:** 2019-07-31

**Authors:** Tingting Hou, Rufeng Zhang, Chongshu Jian, Wanqiu Ding, Yanru Wang, Shukuan Ling, Qi Ma, Xinli Hu, Heping Cheng, Xianhua Wang

**Affiliations:** 1 State Key Laboratory of Membrane Biology, Beijing Key Laboratory of Cardiometabolic Molecular Medicine, Peking-Tsinghua Center for Life Sciences, Institute of Molecular Medicine, Peking University, Beijing, 100871 China; 2 State Key Laboratory of Space Medicine Fundamentals and Application, China Astronaut Research and Training Center, Beijing, 100094 China

**Keywords:** Cell biology, Molecular biology

## Abstract

The impairment of mitochondrial bioenergetics, often coupled with exaggerated reactive oxygen species (ROS) production, is a fundamental disease mechanism in organs with a high demand for energy, including the heart. Building a more robust and safer cellular powerhouse holds the promise for protecting these organs in stressful conditions. Here, we demonstrate that NADH:ubiquinone oxidoreductase subunit AB1 (NDUFAB1), also known as mitochondrial acyl carrier protein, acts as a powerful cardio-protector by conferring greater capacity and efficiency of mitochondrial energy metabolism. In particular, NDUFAB1 not only serves as a complex I subunit, but also coordinates the assembly of respiratory complexes I, II, and III, and supercomplexes, through regulating iron-sulfur biosynthesis and complex I subunit stability. Cardiac-specific deletion of *Ndufab1* in mice caused defective bioenergetics and elevated ROS levels, leading to progressive dilated cardiomyopathy and eventual heart failure and sudden death. Overexpression of *Ndufab1* effectively enhanced mitochondrial bioenergetics while limiting ROS production and protected the heart against ischemia-reperfusion injury. Together, our findings identify that NDUFAB1 is a crucial regulator of mitochondrial energy and ROS metabolism through coordinating the assembly of respiratory complexes and supercomplexes, and thus provide a potential therapeutic target for the prevention and treatment of heart failure.

## Introduction

Being so-called “powerhouses”, mitochondria comprise up to 40% of the heart mass and are central to cardiac bioenergetics.^1,2^ As hazardous by-products of energy metabolism, reactive oxygen species (ROS) are also emitted from the respiratory electron transport chain (ETC), and excessive ROS emission causes oxidative damage to proteins, lipids, and even genetic materials.^3,4^ Studies have shown that >50% of individuals with mutations in genes encoding mitochondrial proteins eventually develop cardiomyopathy,^5^ suggesting that defective cardiac bioenergetics as well as its interlinked ROS metabolism is a fundamental disease mechanism in the heart.^6,7^ As such, building a more robust cellular powerhouse with decreased ROS emission holds the promise for protecting the heart in stressful conditions.

The most important player in mitochondrial energy production is the ETC, which consists of four multi-heteromeric complexes (complexes I–IV) in the inner membrane of the organelle. They catalyze outward proton movement to build a transmembrane electrochemical gradient that drives ATP synthase (complex V) for ATP synthesis.^8^ Individual ETC complexes can also organize into supercomplexes (SCs) of different composition and stoichiometry, which have recently been visualized by cryo-electron microscopy.^9–14^ Such SCs are thought to channel electron transfer more efficiently, limit ROS production, protect vulnerable ETC sites in the complexes from oxidative damage, and stabilize individual complexes.^11,15–21^ Interestingly, SC formation is dynamically regulated in accordance with changes in cellular metabolism,^22–25^ and deficiency of SCs is associated with heart failure.^26^ Therefore, enhancing SC formation might afford an effective therapeutic strategy to make the mitochondria more efficient and safer powerhouses.

NADH:ubiquinone oxidoreductase subunit AB1 (NDUFAB1), also known as mitochondrial acyl carrier protein,^27^ plays a multifunctional role in the organelle. It not only participates in the synthesis of lipoic acid in the type II fatty acid biosynthetic pathway (FAS II),^28,29^ but also acts as an accessory subunit of complex I with a 2:1 stoichiometry.^30^ A recent study has shown that NDUFAB1 interacts with seven mitochondrial LYR motif-containing (LYRM) proteins: LYRM1, LYRM2, LYRM4/ISD11, LYRM5, LYRM7/MZM1L, SDHAF3/ACN9, and FMC1/C7orf55.^31^ Most of them are linked to various mitochondrial metabolic processes, such as the assembly of complex II (for SDHAF3/ACN9) and complex V (for FMC1/C7orf55), deflavination of the electron-transferring flavoprotein (for LYRM5), and biosynthesis of iron-sulfur (FeS) centers (for LYRM4/ISD11).^31^ In yeast cells which lack complex I, Van et al. have shown that NDUFAB1 interacts with and stabilizes the FeS biogenesis complex to regulate the assembly of complexes II and III and SCs.^32^ In addition, previous evidence in 293T cells has also suggested a role of NDUFAB1 in complex I assembly and the existence of a complex I-independent mechanism for *Ndufab1* knockout-induced cell death.^33^ Thus, NDUFAB1 might be a core player in orchestrating mitochondrial energy biogenesis.

In this study, we aimed to test the potential protective role of NDUFAB1 in the mammalian heart using cardiac-specific *Ndufab1* knockout (cKO) and transgenic overexpression (TG) mouse models. We find that NDUFAB1 coordinates the assembly of individual ETC complexes and SCs and acts as a crucial endogenous regulator of the efficiency and capacity of mitochondrial energy metabolism as well as ROS emission. More importantly, NDUFAB1 exerts a cardio-protective effect when the heart is subjected to ischemia-reperfusion (IR) injury. Our findings substantiate that targeting mitochondria for greater bioenergetic capacity and repressed ROS emission with enhanced SC formation is an effective strategy for cardio-protection, and mark NDUFAB1 as a potential therapeutic target for the prevention and treatment of cardiomyopathy.

## Results

### Cardiac-specific ablation of NDUFAB1 causes progressive dilated cardiomyopathy leading to heart failure

**Fig. 1:**
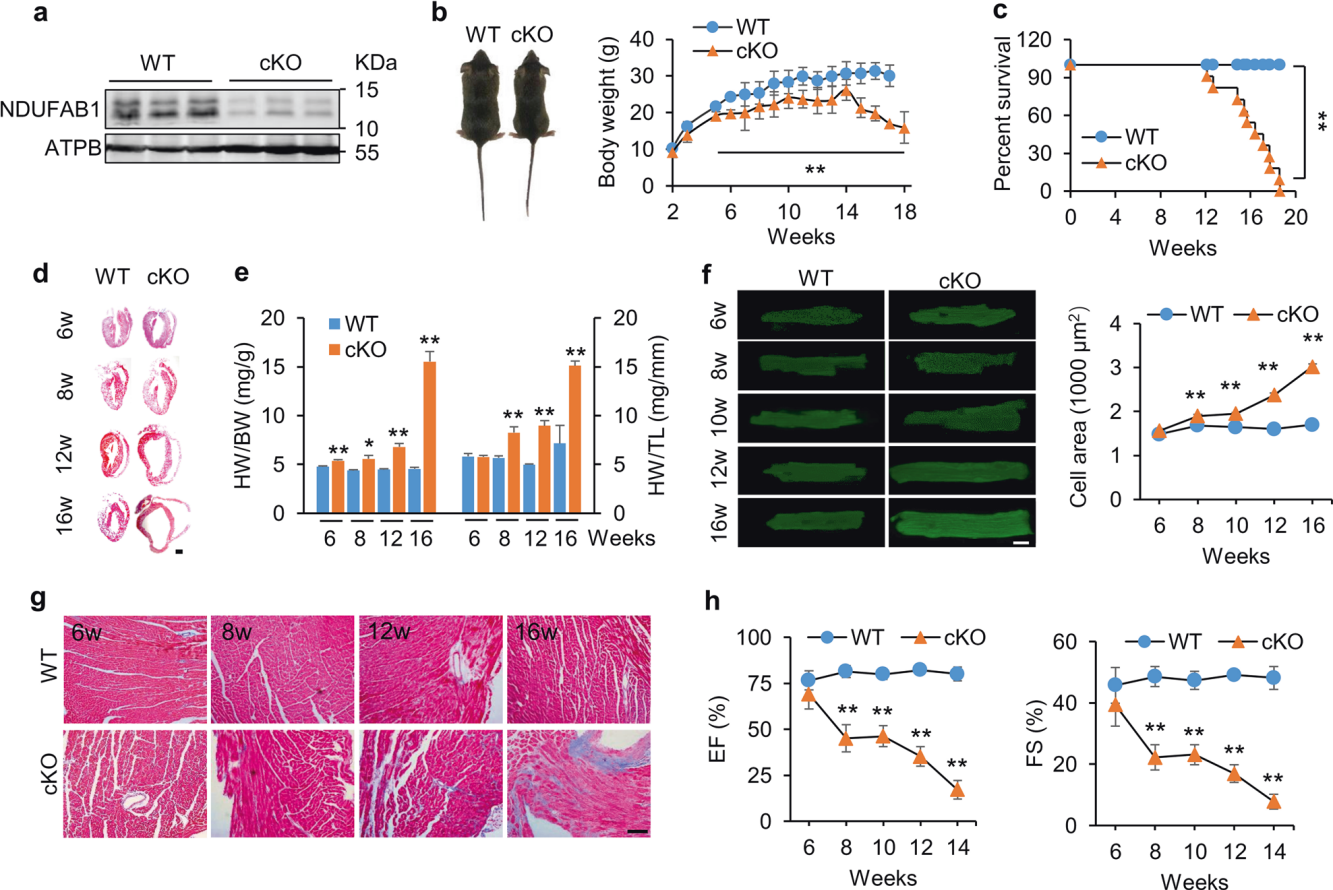
Cardiac-specific knockout of *Ndufab1* (cKO) causes dilated cardiomyopathy leading to heart failure in mice. **a** Western blots of NDUFAB1 in wild-type (WT) and cKO cardiomyocytes. ATPB served as the loading control. **b** Left, photograph of WT and cKO mice at 14 weeks of age. Right, effect of cardiac NDUFAB1 ablation on mouse body weight (mean ± s.e.m.; *n* = 3–16 male mice per group per time point; **p < 0.01 versus WT at the same time point). **c** Kaplan–Meier survival curves for WT and cKO mice (*n* = 11 male mice per group; **p < 0.01 versus WT). **d** Representative longitudinal sections of hearts from male mice at 6–16 weeks (w) old. Scale bar, 5 mm. **e** Ratios of heart weight (HW) to body weight (BW) or tibia length (TL) (mean ± s.e.m.; *n* = 3–14 male mice per group; *p < 0.05, **p < 0.01 versus WT at the same age). **f** Progressive hypertrophy of cardiomyocytes from cKO mice. Left: representative confocal micrographs of cardiomyocytes stained with DCF (scale bar, 20 μm). Right: statistical analysis (mean ± s.e.m.; *n* = 97–611 cells from 4 male mice per group; ** *p* < 0.01 versus WT at the same age). **g** Fibrosis of left ventricular tissue (Masson’s trichrome staining; scale bar, 100 μm). **h** Echocardiographic analysis of cardiac function. EF, ejection fraction; FS, fractional shortening (mean ± s.e.m.; *n* = 3–9 male mice per group; **p < 0.01 versus WT at the same age)

NDUFAB1 was widely expressed in different tissues and was particularly enriched in the heart (Supplementary information, Fig. S1a). Whole-body knockout of *Ndufab1* was embryonic lethal (Supplementary information, Fig. S1b, c), showing that NDUFAB1 is essential for developmental viability. To explore the potential cardiac functions of NDUFAB1, we generated a cardiac-specific knockout mouse model (cKO) wherein exon 3 of *Ndufab1* was flanked by loxP sites (*Ndufab1*^*flox/flox*^, Supplementary information, Fig. S1b). The *Ndufab1*^*flox/flox*^ mice were cross-bred with Mlc2v-Cre mice to allow cardiomyocyte-specific deletion of *Ndufab1*, as previously described.^34^ As shown in Fig. 1a and Supplementary information, Fig. S1d, cardiac expression of *Ndufab1* was decreased by ~90% at the protein level in cKO cardiomyocytes compared to wild-type (WT) cells. The cKO mice were smaller, had a reduced body weight, and even started to lose weight precipitously after 14 weeks of age (Fig. 1b). Meanwhile, the lifespan of cKO mice was markedly shortened, with sudden death beginning at ~12 weeks of age; the maximal lifespan was <19 weeks (Fig. 1c).

We next examined the effect of NDUFAB1 ablation on cardiac morphology and functions at different ages. The cKO hearts progressively enlarged with age and developed dilated cardiomyopathy (Fig. 1d; Supplementary information, Fig. S1e). Compared to WT littermates, the heart weight/body weight ratio in cKO mice was increased by 12.8% at 6 weeks, 26.9% at 8 weeks, 51.5% at 12 weeks, and 241.7% at 16 weeks; and the heart weight/tibia length ratio was not significantly altered at 6 weeks and increased by 45.5% at 8 weeks, 80.1% at 12 weeks, and 110.9% at 16 weeks (Fig. 1e). The slight increase of heart weight/body weight ratio without change of heart weight/tibia ratio at 6 weeks is due to mild reduction of body weight (Fig. 1b). At the cellular level, the longitudinal-section area of cardiomyocytes was nearly constant in WT but increased progressively in cKO mice, with no significant change at 6 weeks and reaching a 78% increase at 16 weeks (Fig. 1f), indicative of cardiomyocyte hypertrophy. Masson staining showed that NDUFAB1 ablation induced extensive cardiac fibrosis early at 8 weeks (Fig. 1g; Supplementary information, Fig. S1f). Consistent with the changes of cardiac morphology, echocardiography revealed that the ejection fraction (EF) and fractional shortening (FS) of cKO hearts were unaltered at 6 weeks, but halved at 8 weeks; and the EF was diminished by 79% and FS by 84% at the age of 14 weeks (Fig. 1h), while the heart rate remained unchanged (Supplementary information, Fig. S2a). The thickness of both the left ventricular posterior wall and the inter-ventricular septum was decreased whereas the left ventricular diameter and volume were increased with age in cKO hearts (Supplementary information, Fig. S2b, c). These findings indicate that cardiac-specific ablation of NDUFAB1 leads to dilated cardiomyopathy accompanied by cardiomyocyte hypertrophy, interstitial fibrosis, and systolic and diastolic dysfunction, eventually resulting in heart failure and sudden death, revealing an essential role of NDUFAB1 in cardiac function.

### Mitochondrial dysfunctions in *Ndufab1* cKO heart

**Fig. 2:**
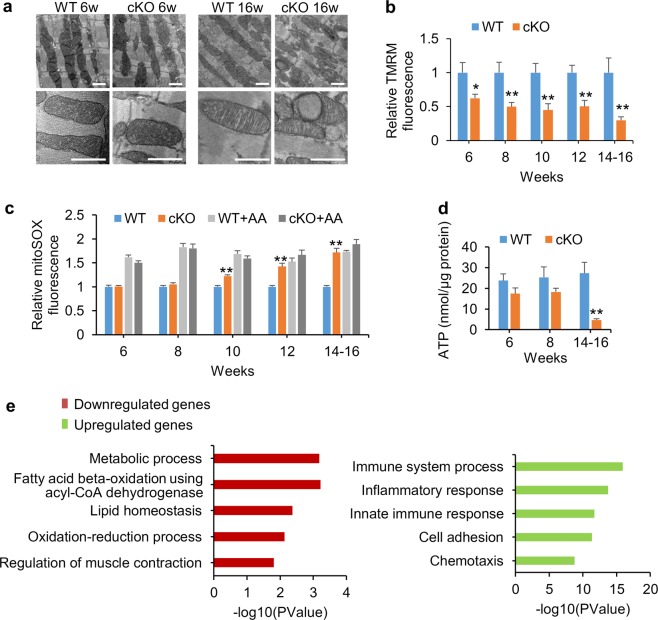
Defective mitochondrial bioenergetics and ROS metabolism in *Ndufab1* cKO mice. **a** Electron micrographs of mitochondria in left ventricular tissue from 6- and 16-week-old male mice (scale bars, 1 μm). **b** Decline of ΔΨ_m_ in cKO myocardium assessed by TMRM staining and confocal imaging of isolated cardiomyocytes. The intensity of FCCP-sensitive TMRM fluorescence was calculated and the intensity in cKO group was normalized to that of WT at the same age (*n* = 13–26 cells from three male mice per group; *p < 0.05, **p < 0.01 versus WT). **c** Progressive elevation of mitochondrial ROS measured with mitoSOX in isolated cardiomyocytes. Antimycin A (AA, 5 μg/mL) was used to induce massive ROS production. The intensities of mitoSOX fluorescence in the groups of cKO, WT + AA and cKO + AA were normalized to that of WT at the same age (*n* *=* 31–179 cells from 4–5 male mice per group; ** *p* < 0.01 versus WT). **d** ATP levels of isolated cardiomyocytes measured with luciferin assay (*n* = 3–4 male mice per group; ** *p* < 0.01 versus WT). **e** Top 5 enriched Gene Ontology terms of downregulated and upregulated genes. RNA-seq analysis was performed on cKO and WT hearts at 8–16 weeks (*n* = 4 male mice per group) and the genes upregulated or downregulated were analyzed

To investigate the mechanism underlying the cardiomyopathy and heart failure induced by NDUFAB1 ablation, we next assessed changes of cardiac mitochondrial functions at different ages of cKO mice. Electron microscopic analysis revealed that cardiac mitochondria were in disarray along the sarcomeres with some exhibiting irregularities in the cristae formation in 16-week-old cKO mice, whereas mitochondrial morphology was apparently normal at 6 weeks, when the cardiac function hadn’t been impaired yet (Fig. 2a). The mitochondrial DNA content and cross-sectional area of individual mitochondria didn’t display detectable changes whereas the total mitochondrial volume fraction and mitochondrial number were slightly increased in 16-week-old cKO mice (Supplementary information, Fig. S3). Measuring mitochondrial membrane potential (ΔΨ_m_) in isolated cardiomyocytes with the potential-sensitive fluorescent probe tetramethyl rhodamine methyl ester (TMRM) showed that NDUFAB1 ablation significantly decreased ΔΨ_m_ even at 6 weeks (Fig. 2b) when the heart morphology and function were normal (Fig. 1), indicating mitochondrial dysfunction is an early event in cKO mice preceding the onset of cardiomyopathy. Measurements with the fluorescent probe mitoSOX showed that mitochondrial ROS level was significantly elevated in cKO cardiomyocytes compared to WT at 10 weeks, and increased by 72% at 14–16 weeks, reaching a level comparable to that induced by 5 μg/mL antimycin A (Fig. 2c). The cellular ATP level, as measured with the luciferin luminescence assay, showed a trend of decrease in cKO mice from 6 to 8 weeks old and was significantly lowered in 14–16-week or older cKO mice (Fig. 2d). This result suggests that, while the ATP level is initially tightly safeguarded in the heart,^35–39^ NDUFAB1 ablation curtails the energy reserve capacity, exhaustion of which impairs ATP homeostasis. Taken together, these results indicate that NDUFAB1 is essential for mitochondrial bioenergetics and ROS metabolism.

Next, we analyzed the transcriptome of left ventricles from cKO and WT mice by RNA sequencing (RNA-seq). Among 11494 genes analyzed, 430 were downregulated and 1081 were upregulated (Supplementary information, Tables S1 and S3). Interestingly, the top 5 Gene Ontology terms of the downregulated genes were mainly associated with mitochondrial metabolism, including metabolic process, fatty acid metabolism, lipid homeostasis, oxidation-reduction process (Fig. 2e; Supplementary information, Table S2), in good agreement with the bioenergetic defects in the cKO heart (Fig. 2b–d). The downregulated genes were involved in the regulation of muscle contraction, consistent with the phenotype of cardiomyopathy (Fig. 2e). Meanwhile, the top enriched Gene Ontology terms of upregulated genes were mostly involved in immune system process, inflammatory response, cell adhesion, and chemotaxis (Fig. 2e; Supplementary information, Table S4). We deduce that these upregulated processes might reflect compensatory as well as maladaptive responses to cardiac damage due to mitochondrial dysfunctions.

### Impaired assembly of mitochondrial ETC complexes and SCs in cKO hearts

**Fig. 3:**
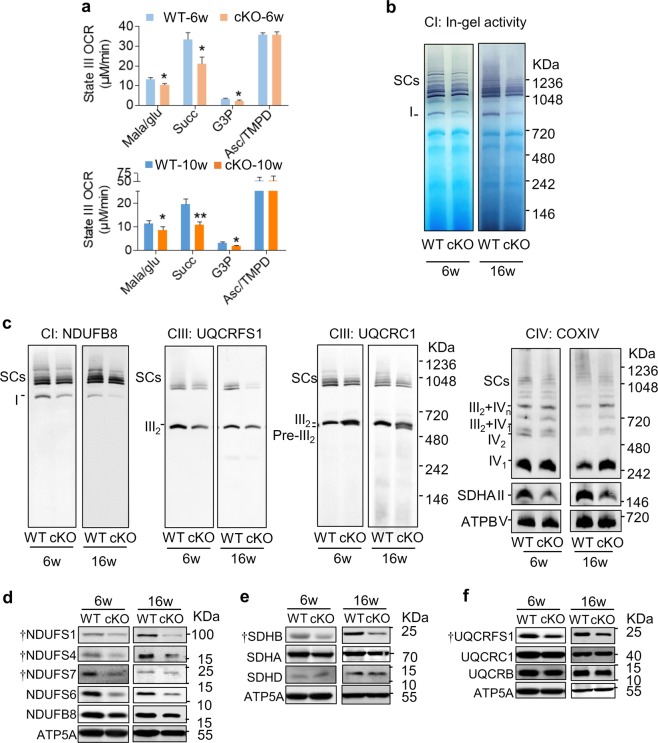
Defective respiration and impaired assembly of ETC complexes I-III and SCs in cKO hearts. **a** Effect of NDUFAB1 ablation on state III oxygen consumption rate (OCR) in isolated mitochondria at the age of 6 or 10 weeks. For each measurement, 100 μg mitochondria were used. Different respiratory substrates were used: malate/glutamate (Mala/glu, 5 mM) for complex I, succinate (Succ, 5 mM) for complex II, glycerol-3-phosphate (G3P, 5 mM) for complex III, and ascorbate (Asc, 2.5 mM)/N,N,N’,N’-tetramethyl-p-phenylenediamine (TMPD, 0.5 mM) for complex IV, and ADP (100 μM) (mean ± s.e.m.; *n* = 3–14 male mice of per group; *p < 0.05, **p < 0.01 versus WT at the same age). **b** In-gel activity of SCs and complex I at the age of 6 or 16 weeks. **c** BN-PAGE immunoblots of individual ETC complexes and SCs at the age of 6 or 16 weeks. SCs were visualized by antibodies against subunits of complex I (CI, NDUFB8), complex III (CIII, UQCRFS1 or UQCRC1), and complex IV (CIV, COX IV). Complex II was visualized by antibody against SDHA, and complex V with antibody against ATPB. **d–f** Western blots of subunits of complex I (**d**), complex II (**e**), and complex III (**f**) at the age of 6 or 16 weeks. Dagger represents FeS-containing subunits. ATP5A served as the loading control

To further understand the mechanism underlying these mitochondrial energy and ROS metabolism dysfunctions induced by NDUFAB1 ablation, we tested whether NDUFAB1 deficiency affected lipoic acid synthesis in FAS II pathway. We measured pyruvate dehydrogenase activity as lipoic acid is its obligate cofactor,^40^ and found similar activities in WT and cKO hearts (Supplementary information, Fig. S4a). Further, the protein lipoylation status was not significantly altered in cKO hearts (Supplementary information, Fig. S4b). These results revealed NDUFAB1 ablation did not affect FAS II pathway in the heart. Given the pivotal role of the mitochondrial ETC in energy metabolism and ROS production, we next investigated whether and how NDUFAB1 ablation impacted on the assembly and activity of individual ETC complexes in the heart. First, we measured the respiratory activity of isolated mitochondria from 6-week-old mice when the cardiac morphology and function were normal and 10-week-old mice when cardiomyopathy was already developed. In the presence of substrates of either complex I, complex II or complex III, the oxygen consumption rates (OCRs) of cKO mitochondria were significantly decreased at both ages (Fig. 3a; Supplementary information, Fig. S5a–c), whereas the OCR supported by complex IV substrate remained unchanged (Fig. 3a; Supplementary information, Fig. S5d). These data suggest that, rather than being merely a complex I subunit, NDUFAB1 alters the ETC function at multiple sites.

Further, we assessed the assembly of ETC complexes using blue native polyacrylamide gel electrophoresis (BN-PAGE). Consistent with previous reports,^41,42^ complexes I, III, and IV were assembled into SCs, as evidenced by immunoblot analysis following BN-PAGE using antibodies against NDUFB8 (complex I), SDHA (complex II), UQCRFS1 or UQCRC1 (complex III), COXIV (complex IV), and ATPB (complex V), while complexes II and V mainly remained as individual entities in both WT and cKO groups (Fig. 3c). Moreover, NDUFAB1 ablation decreased the SCs comprised of complexes I, III, and IV in the hearts at both ages (Fig. 3c; Supplementary information, Fig. S6b). We also assessed the abundance of individual complexes and found that complexes I, II, and III were significantly diminished in cKO mitochondria at both ages (Fig. 3c; Supplementary information, Fig. S6b), consistent with the aforementioned functional data (Fig. 3a). The level of individual complex IV was increased in cKO mitochondria due to decreased SC assembly (Fig. 3c; Supplementary information, Fig. S6b). Further analysis showed the percentages of complex III- and complex IV-containing SCs were decreased in cKO mitochondria at both ages (Supplementary information, Fig. S6c). The percentage of complex I-containing SCs was not significantly changed because both the free and complexed complex I were proportionally diminished in cKO mitochondria (Supplementary information, Fig. S6b, c). In-gel activity analysis in the presence of complex I substrate revealed that the activity of either complex I or SCs was markedly lowered in the absence of NDUFAB1 (Fig. 3b; Supplementary information, Fig. S6a). Moreover, we measured the abundance of individual complexes and SCs in the hearts of newborn mice (born within 24 h) and found that complexes I, II, III and SCs were diminished while complex IV was not significantly changed in cKO mitochondria (Supplementary information, Fig. S7b, d). Likewise, in-gel activity analysis in the presence of complex I substrate showed decreased activities of complex I and SCs in cKO mitochondria of newborn mice (Supplementary information, Fig. S7a, c). Meanwhile, knocking down *Ndufab1* in cultured neonatal rat cardiomyocytes also decreased the abundance of complex I and SCs (Supplementary information, Fig. S8). All these results implicate that NDUFAB1 is essential for the assembly of complexes I, II, III, and SCs in the heart. Furthermore, the early onset of impaired mitochondrial energetics induced by disrupted assembly of ETC complexes and SCs indicates a causative role of this NDUFAB1 ablation-induced ETC defect in heart failure.

By measuring the abundance of individual subunits of complexes I-III, we found that the FeS-containing subunits, SDHB (succinate dehydrogenase subunit B) of complex II and UQCRFS1 (ubiquinol-cytochrome c reductase, Rieske iron-sulfur polypeptide 1) of complex III, were both significantly decreased, whereas the other subunits examined were unaltered in the absence of NDUFAB1 (Fig. 3e, f; Supplementary information, Fig. S6e). This indicates that NDUFAB1 ablation selectively disrupts FeS-containing subunits of complexes II and III. Concomitantly, a complex III assembly intermediate lacking UQCRFS1, which is incorporated into complex III at the last step,^43^ accumulated in cKO mitochondria (Fig. 3c; Supplementary information, Fig. S7b). These findings suggest that NDUFAB1 coordinates the assembly of ETC complexes and SCs through regulating FeS cluster biogenesis as reported previously in yeast cells lacking complex I.^32^ In this regard, we found that ISCU and NFS1, two important components of the FeS biogenesis complex,^44,45^ were down-regulated in cKO cardiomyocytes (Supplementary information, Fig. S9). For complex I, however, all subunits examined, regardless of whether they contain (NDUFS1, NDUFS4, and NDUFS7) or don’t contain (NDUFS6 and NDUFB8) FeS cluster(s), were dramatically decreased in cKO mitochondria (Fig. 3d; Supplementary information, Fig. S6d), indicating the involvement of additional mechanism by which NDUFAB1 regulates its assembly (see Discussion). Since the mRNA levels of the above subunits of complexes I–III were not significantly altered (Supplementary information, Fig. S10), the NDUFAB1-mediated regulation appears to occur at the protein rather than the mRNA level, presumably by affecting protein stability. Therefore, NDUFAB1 coordinates the assembly of complexes I–III and SCs through its dual roles both as a regulator of FeS biogenesis and as a complex I subunit.

Different from the effect of NDUFAB1 ablation on respiratory complexes, knockout of *Ndufs4*, another accessory subunit of complex I, induced complex I deficiency but showed little effect on other complexes.^46^ Meanwhile, NDUFS4 ablation led to decreased NAD^+^/NADH ratio and increased protein acetylation,^46^ however, we found that neither the NAD^+^/NADH ratio nor protein acetylation levels were significantly altered in *Ndufab1* cKO cardiomyocytes (Supplementary information, Fig. S11). These results suggest a specific role of NDUFAB1 in the assembly of respiratory complexes and SCs. In addition, mitochondrial cardiolipin content was not altered in *Ndufab1* cKO mitochondria (Supplementary information, Fig. S12). All these results substantiate that NDUFAB1 ablation-induced cardiomyopathy is mainly caused by impaired assembly of ETC complexes and SCs.

### Enhanced mitochondrial functions in *Ndufab1* transgenic hearts

**Fig. 4:**
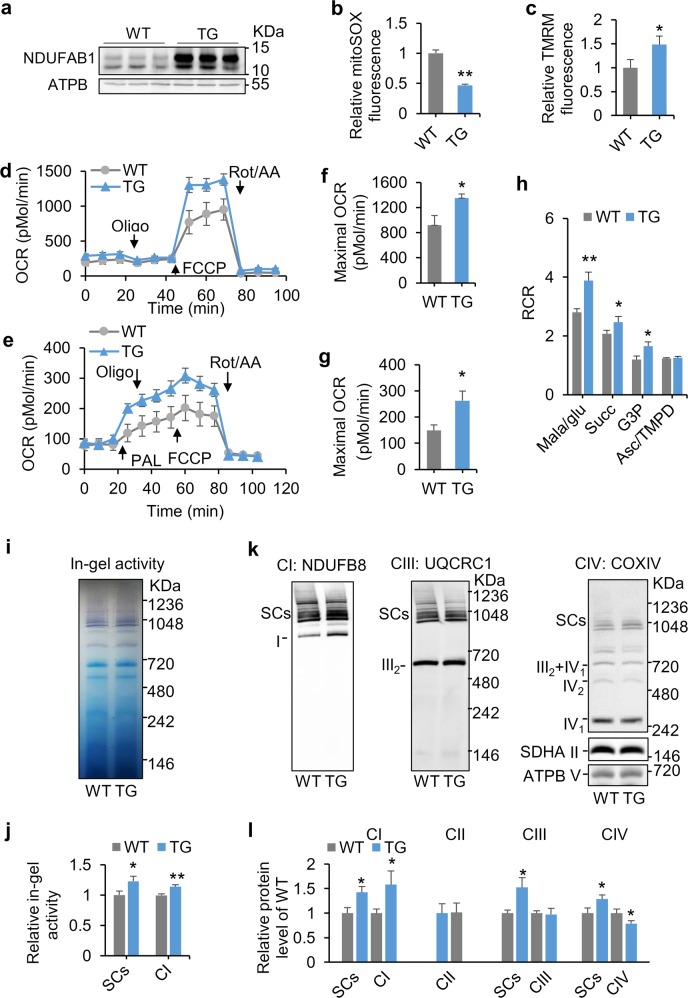
Enhanced mitochondrial bioenergetics and SC assembly in the heart of *Ndufab1* transgenic mice (TG). TG and WT littermates of 2–4 months old were used. **a** Western blots for NDUFAB1 expression in WT and TG hearts. ATPB served as the loading control. **b** Decreased mitochondrial ROS level in TG cardiomyocytes. The intensity of mitoSOX fluorescence of TG was normalized to that of WT (*n* *=* 34–41 cells from 3 male mice; **p < 0.01 versus WT). **c** Increased ΔΨ_m_ in TG cardiomyocytes. The intensity of FCCP-sensitive TMRM fluorescence of TG was normalized to that of WT (*n* *=* 12–15 cells from three male mice per group; *p < 0.05 versus WT). **d**, **e** Whole-cell OCR measured with Seahorse using glucose/pyruvate (**d**) or palmitate (**e**) as the substrate. Arrows indicate the time of adding palmitate (PAL, 200 µM), oligomycin (Oligo, 1 µM), FCCP (0.5 µM), and rotenone/antimycin A (Rot/AA, 1 µM for each). **f**, **g** Statistical analysis of maximal OCR in **d** and **e** (mean ± s.e.m.; *n* *=* 10–13 wells from 3 male mice per group; *p < 0.05 versus WT). **h** Changes of respiratory control ratio (RCR) with different substrates in isolated mitochondria (mean ± s.e.m.; *n* *=* 3–8 male mice per group; *p < 0.05, **p < 0.01 versus WT). **i** In-gel activity of SCs and complex I. **j** Statistical analysis of **i**. The activity of TG was normalized to WT (mean ± s.e.m.; *n* = 5 male mice per group; *p < 0.05, **p < 0.01 versus WT). **k** BN-PAGE immunoblots of SCs and individual ETC complexes as in Fig. 3c. **l** Statistical analysis of **k**. The expression level of TG complexes and supercomplexes was normalized to that of WT (mean ± s.e.m.; *n* *=* 4–8 male mice per group; *p < 0.05 versus WT)

The results thus far reveal that NDUFAB1 plays an essential role in coordinating the dynamic assembly of individual ETC complexes and SCs and thereby regulates mitochondrial bioenergetics and ROS metabolism. We reckoned that augmenting the abundance of NDUFAB1 might provide an effective strategy to build a more robust and efficient powerhouse to benefit the heart, providing that endogenous NDUFAB1 is present at a sub-saturating level. To test this possibility, we generated an *Ndufab1* transgenic mouse model (TG) using a β-actin promoter (Supplementary information, Fig. S13a) in which NDUFAB1 was overexpressed ~8-fold in the heart (Fig. 4a; Supplementary information, Fig. S13b). Compared with WT littermates, TG mice grew normally and had similar lifespans under our experimental conditions (Supplementary information, Fig. S13c, d). Cardiac morphology and functional performance determined by echocardiography (Supplementary information, Fig. S13e–g), mitochondrial morphology revealed by electron microscopy (Supplementary information, Fig. S13h–j), and mitochondrial DNA content (Supplementary information, Fig. S13l) were all comparable in WT and TG hearts. Nonetheless, the mitochondrial ROS level was markedly decreased in TG cardiomyocytes (Fig. 4b), suggesting an alleviation of basal oxidative stress. The ΔΨ_m_ was significantly increased (Fig. 4c), and the maximal OCR with either glucose/pyruvate or palmitate as substrate measured by Seahorse assay was higher in TG than in WT cardiomyocytes (Fig. 4d–g). These results imply an enhanced reserve capacity for ATP production, although the homeostatic ATP level was unaltered (Supplementary information, Fig. S13k). The respiratory control ratio was significantly improved in the presence of substrates of complex I, II, or III in TG cardiac mitochondria (Fig. 4h), indicating greater efficiency of mitochondrial energy metabolism. Moreover, the electron flow assay showed enhanced activities of complexes I, II and III in TG mitochondria (Supplementary information, Fig. S14), indicating that NDUFAB1 overexpression augments the ETC activity.

At the molecular level, in-gel activity analysis showed that activity of both complex I and SCs were significantly enhanced in TG hearts (Fig. 4i, j), and BN-PAGE analysis showed that NDUFAB1 overexpression significantly increased the contents of complex I and SCs (Fig. 4k, l; Supplementary information, Fig. S15a), whereas the expression of all subunits examined, including those for complexes I-III, was unchanged (Supplementary information, Fig. S15b, c). Importantly, overexpression of NDUFAB1 in cultured neonatal cardiomyocytes enhanced the abundance of complex I and SCs without significant effect on the other complexes (Supplementary information, Fig. S16). In addition, NDUFAB1 overexpression did not impact on FAS II pathway in the heart, because neither pyruvate dehydrogenase activity nor protein lipoylation status was significantly altered in TG mitochondria (Supplementary information, Fig. S17). The NAD^+^/NADH ratio and protein acetylation levels were also comparable in the WT and TG hearts (Supplementary information, Fig. S18). Taken together, NDUFAB1 overexpression enhances the abundance of complex I and SCs, conferring on mitochondria greater capacity and efficiency of energy metabolism and less ROS emission.

### NDUFAB1 overexpression protects the heart against IR injury

**Fig. 5:**
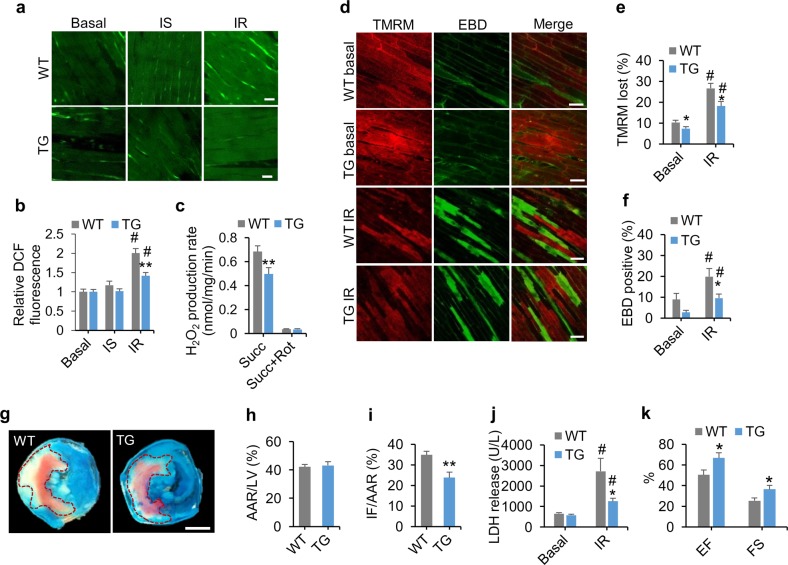
NDUFAB1 overexpression protects the heart against IR injury. TG and WT male littermates of 2–4 months old were used. **a** Alleviated myocardial ROS production in TG hearts. Confocal images of DCF-stained myocardium after Langendorff perfusion and IR injury. Ischemia (IS) was mimicked by stopping perfusion. See Supplementary information, Fig. S19a for experimental protocol. Scale bars, 20 μm. **b** Statistical analysis of **a** (mean ± s.e.m.; *n* *=* 32–51 image frames from 4–7 male mice per group; **p < 0.01 versus WT, ^#^*p* < 0.01 after IR versus basal). **c** Rotenone (Rot)-sensitive H_2_O_2_ production at complex I in WT and TG cardiac mitochondria (mean ± s.e.m.; *n* *=* 6 male mice per group; **p < 0.01 versus WT). Succinate (Succ, 5 mM) was used as the substrate of complex II and H_2_O_2_ was measured with Amplex red. **d** Images for simultaneous measurement of ∆Ψ_m_ (TMRM, pseudocolor: red) and cardiomyocyte death (EBD uptake, pseudocolor: green) in Langendorff-perfused hearts subjected to IR injury. Note that all cells with intact ∆Ψ_m_ are EBD-negative, and all EBD-positive cells display loss of ∆Ψ_m_, but not vice versa. Scale bars, 50 μm. **e, f** Percentages of cells with loss of ∆Ψ_m_ (**e**) and EBD-positive cells (**f**) (mean ± s.e.m.; *n* = 9–10 male mice per group; *p < 0.05 versus WT, ^#^*p* < 0.01 after IR versus basal). **g** Representative cross-sections of WT and TG hearts after IR injury. See Supplementary information, Fig. S19b for the experimental protocol. White, infarcted area (IF); red, rest of the area at risk (AAR); blue, tissue not at risk. The AAR was demarcated by dotted line. Scale bar, 1 mm. **h, i** Statistical analysis of AAR (**h**) and IF (**i**) reported as the ratios of AAR to left ventricular area (LV) and IF to AAR (*n* *=* 5–8 male mice per group; **p < 0.01 versus WT). **j** Measurement of serum LDH release after IR (*n* *=* 10–13 male mice per group; *p < 0.05 versus WT, ^#^*p* < 0.01 after IR versus basal). **k** Echocardiographic analysis of cardiac function 48 h after IR injury. EF, ejection fraction; FS, fractional shortening (mean ± s.e.m.; *n* = 4 male mice per group; *p < 0.05 versus WT)

The cardiac performance as well as the lifespan of TG mice was similar to that of WT littermates under unchallenged conditions (Supplementary information, Fig. S13). However, TG mice might be more resistant to cardiac injury because of augmented capacity and efficiency of mitochondrial bioenergetics along with attenuated ROS production. Next, we subjected the WT and TG hearts to IR injury to unmask this potential cardio-protective role of NDUFAB1. First, we applied an ex vivo IR protocol based on Langendorff perfusion with 30-min ischemia followed by 30-min reperfusion (Supplementary information, Fig. S19a). Since excessive ROS production during IR is a major contributor to IR injury,^47–49^ we tracked the ROS production with indicator 5-(and-6)-chloromethyl-2′,7′-dichlorodihydrofluorescein diacetate acetyl ester (DCF) (Fig. 5a). In WT hearts, there was a trend of increasing ROS during ischemia followed by a prominent ROS burst immediately after reperfusion (Fig. 5a, b). Remarkably, the ROS burst after IR was ameliorated by 29% in TG hearts (Fig. 5a, b). Furthermore, we measured H_2_O_2_ production in the presence of succinate in isolated cardiac mitochondria using the Amplex Red assay^50^ and found that the rotenone-sensitive H_2_O_2_ production was significantly attenuated in TG mitochondria (Fig. 5c).

Because loss of ΔΨ_m_ not only manifests a defective bioenergetics but also is an early event of cell death,^51,52^ we further assessed cardiac damage by monitoring loss of ΔΨ_m_ with TMRM and membrane integrity with evans blue dye (EBD). Our results showed that cells that both lost TMRM and were EBD-positive were significantly fewer in TG than WT hearts (Fig. 5d–f). Moreover, we adopted an in vivo IR experimental protocol in which a 30-min ischemia was followed by 24-h reperfusion (Supplementary information, Fig. S19b). In agreement with the ex vivo IR results, the area of infarction in TG was significantly smaller than that in WT hearts (Fig. 5g–i). Meanwhile, lactate dehydrogenase (LDH) release induced by IR injury was significantly attenuated in TG mice (Fig. 5j). Echocardiography revealed that TG mice exhibited significantly improved heart function at 48 h after IR injury compared to WT mice (Fig. 5k). Altogether, these findings demonstrate that increasing the abundance of mitochondrial NDUFAB1 attenuates ROS production and protects the heart against IR injury.

## Discussion

The heart is quite sensitive to mitochondrial dysfunction such that most individuals with mutated mitochondrial proteins eventually develop cardiomyopathy.^5^ Not only are mitochondria the predominant powerhouses of the heart, supplying more than 90% of the ATP,^2^ but also excessive mitochondrial ROS are the culprits in many oxidative heart diseases including IR injury. Here we have shown, for the first time, that NDUFAB1 is a key endogenous regulator of mitochondrial bioenergetics and ROS metabolism in the mammalian heart and a cardio-protector when the heart is subjected to stress and injury. Our ex vivo and in vivo experimental results have established the essential role of NDUFAB1 in the maintenance of normal cardiac functions and the cardio-protective effects of NDUFAB1 overexpression. In the cKO heart, NDUFAB1 ablation led to lowered ΔΨ_m_ and diminished ATP production. Meanwhile, ROS emission was elevated to pathologically high levels, aggravating the situation of insufficient energy supply. Cardiac performance deteriorates precipitously once the energy reserve is exhausted and the ATP level can no longer be held constant. Decompensatory myocardial remodeling also ensues, manifested as myocardial fibrosis, cardiomyocyte hypertrophy, and dilation of the ventricles with thinning of the ventricular walls, rapidly leading to heart failure and sudden death. Conversely and more importantly, increasing NDUFAB1 abundance makes the mitochondria not only more robust, featured with augmented energy reserve capacity and efficiency, but also safer in terms of repressed ROS emission.

Both the improved bioenergetics and the diminished ROS production appear to be indispensable for NDUFAB1-mediated cardio-protection. On one hand, as the heart is highly demanding of energy, increasing the energy reserve capacity would make the heart more resistant to insults that tend to curtail ATP production. On the other hand, since excessive ROS constitute a crucial driving factor for IR injury in the heart,^47–49^ repression of basal ROS, and particularly the ROS burst during IR, should be beneficial. Specifically, we have found that NDUFAB1 overexpression represses ROS production at the onset of reperfusion after ischemia. In agreement, our unpublished data showed that NDUFAB1 was decreased in human failing hearts and in mouse hearts after IR, supporting the possibility that NDUFAB1 desregulation may contribute to mitochondrial dysfunction observed in heart failure and IR patients. Altogether, our results substantiate the emerging concept that energetically more robust and ROS-repressed mitochondria can protect the heart in stressful situations.

Enhanced assembly of respiratory SCs induced by NDUFAB1 overexpression contributes to improved bioenergetic capacity and efficiency reflected by increased mitochondrial membrane potential and respiration and repressed ROS production. Formation of SCs has been shown to enhance the catalytic activity of individual components,^53^ facilitate electron transfer, and reduce ROS production.^17,22,41^ It has been recently reported that accumulated succinate during ischemia fuels ROS burst at complex I through reverse electron transport.^54^ In the TG hearts, however, even though the complex I content was increased, both reverse electron transport and succinate accumulation might be inhibited by enhanced SC formation and heightened respiration, decreasing IR-induced ROS production. Conversely, NDUFAB1 ablation caused impaired SC formation, leading to deficiency of mitochondrial respiration and individual ETC complex activity. Elevated mitochondrial ROS levels were also observed in cKO cardiomyocytes. Thus, our findings implicate that enhancing SC formation can be an effective strategy to enhance mitochondrial bioenergetics and limit ROS production.

NDUFAB1 coordinates the assembly of ETC complexes and SCs, mainly by regulating the biogenesis of FeS clusters as reported previously.^32^ In the absence of NDUFAB1, the assembly of complexes I-III, but not complex IV, was severely impaired. This result is in good agreement with the fact that complexes I-III all contain FeS clusters, but complex IV does not.^8^ Furthermore, only the FeS-containing subunits of complexes II and III were selectively down-regulated, and the premature complex III lacking the FeS-containing subunit UQCRFS1 accumulated in cKO mitochondria. For complex I, however, an additional mechanism must be invoked because all subunits examined, including those lacking FeS clusters, were downregulated in the absence of NDUFAB1. When NDUFAB1 was upregulated, greater effects were found on the assembly of complex I than complex II or III. These qualitative and quantitative differences are not unexpected, because cryo-electron microscopy has identified two copies of NDUFAB1 in complex I, and these are critically positioned for inter-subunit interactions within the complex.^55^ Therefore, NDUFAB1 upregulation enhances complex I assembly presumably through stabilizing inter-subunit interactions. The enhanced complex I then promotes SC formation in TG mitochondria, in agreement with the proposed model that complex I assembly is completed before SC formation.^56,57^ Altogether, it is possible that NDUFAB1 modulates SC formation indirectly by regulating the assembly of individual complexes I-III, because it has been suggested that SCs form after complete assembly of the individual complexes.^11,33,56^ Meanwhile, our data also suggest the alternative possibility that NDUFAB1 participates directly in the assembly of SCs. If this is the case, NDUFAB1 might reversely regulate the abundance of individual complexes by affecting SC formation, since SCs have been suggested to stabilize individual complexes.^16,19–21^

In summary, we demonstrate that NDUFAB1 plays an essential role in mitochondrial bioenergetics and cardiac function through coordinating the assembly of respiratory complexes and SCs. Mechanistically, these effects are mediated mainly by its dual roles both as a regulator of FeS biogenesis and as a complex I subunit. Augmenting NDUFAB1 abundance is sufficient to enhance the energy metabolism efficiency and reserve capacity of mitochondria, and to lower ROS production. It also protects the heart against injurious insults. Given recent cryo-EM evidence for respiratory SCs,^9–14^ the present study underscores the dynamic regulation of SC formation and reveals the possibility to target SC formation as a means to build stronger and safer powerhouse and hence stronger heart. Overall, we identify a novel target to build a more robust mitochondria powerhouse for a stronger heart. It is thus tempting to speculate that targeting NDUFAB1 to promote ETC complex and SC assembly might afford a potential therapeutic strategy for the prevention and treatment of mitochondrial bioenergetics-centered heart diseases.

## Materials and methods

### Study approval

All animal experiments were carried out following the rules of the American Association for the Accreditation of Laboratory Animal Care International and the Guide for the Care and Use of Laboratory Animals published by the US National Institutes of Health (NIH Publication No. 85-23, revised 1996). All procedures were approved by the Animal Care Committee of Peking University accredited by AAALAC International (IMM-ChengHP-14).

### Generation of *Ndufab1* knockout and transgenic mice

Floxed *Ndufab1* mice were generated by standard techniques using a targeting vector containing a neomycin-resistance cassette flanked by FRT sites. Briefly, exon 3 of the *Ndufab1* gene was inserted into two flanking LoxP sites. After electroporation of the targeting vector into embryonic stem (ES) cells v6.5 (129 × C57), G418-resistant ES cells were screened for homologous recombination by Southern blot. Two heterozygous recombinant ES clones were identified and microinjected into blastocysts from C57BL/6 J mice to generate germline-transmitted floxed heterozygous mice (*Ndufab1*^*f/+*^). Homozygous Ndufab1-floxed mice (*Ndufab1*^*f/f*^) were obtained by inbreeding the *Ndufab1*^*f/+*^ mice.

Cardiac-specific *Ndufab1* knockout mice were generated as previously described.^34^ Briefly, *Ndufab1*^*f/f*^ mice were bred with Mlc2v-Cre mice in which Cre recombinase expression was controlled by the myosin light chain 2v promoter to generate double heterozygous Mlc2v-Cre and *Ndufab1* floxed mice (Mlc2v-Cre/ *Ndufab1*^*f/+*^). The mice were then backcrossed with homozygous *Ndufab1*^*f/f*^ mice to generate Mlc2v-Cre^+^/*Ndufab1*^*f/f*^ cardiac-specific *Ndufab1* knockout mice and Mlc2v-Cre^−^/*Ndufab1*^*f/f*^ littermate controls. Mice were genotyped by PCR analysis using tail DNA, and flox primers (forward, ACAAATTCTCCCTGATGTCCTT; reverse, TGTTCAACTTCATTTTGAGGTGGT) and Cre primers (forward, GCGGTCTGGCAGTAAAAACTATC; reverse, GTGAAA CAGCATTGCTGTCACTT) were used.

To generate global *Ndufab1* knockout mice, *Ndufab1*^*f/f*^ mice were bred with Prm-Cre mice in which Cre recombinase expression was controlled by the mouse protamine 1 promoter in the male germ line to generate heterozygous Prm-Cre and *Ndufab1* floxed mice (Prm-Cre^+^/*Ndufab1*^*f/+*^). The male mice were bred with wild-type (WT) C57BL/6 J females to generate heterozygous *Ndufab1*^*+/−*^ mice. The heterozygous mice were intercrossed to generate homozygous *Ndufab1*^*−/−*^ mice. Mice were genotyped by PCR analysis using tail DNA, and WT primers (forward, ACAAATTCTCCCTGATGTCCTT; reverse, ACTGTATTCATGCCAGCATGTG) and KO primers (forward, ACAAATTCTCCCTGATGTCCTT; reverse, TCTGTTGTGCCCAGTCATAG) were used.

To generate pan-tissue transgenic mice expressing *Ndufab1*, the *Ndufab1* cDNA was cloned into the pUCCAGGS vector downstream of the chicken β-promoter. The construct was linearized with *Hin*dIII and *Pvu*I to release the transgenic cassette, purified with a DNA purification kit (Qiagen), and microinjected into fertilized eggs of C57BL/6J mice. The mice were genotyped by PCR analysis using the primers AGCCTCTGCTAACCATGTTC (forward) and GTCCAAACTGTCTAAGCCCA (reverse).

The mice were housed under a 12-h-light cycle; food and water were provided ad libitum.

### Echocardiography

Mice were anesthetized with isoflurane (2% in 100% O_2_ at 0.5 L/min) or avertin (2.5%, 0.01 mL/g, intraperitoneally, i.p.) and transthoracic echocardiography was performed using a VEVO-2100 Imaging System (Visual Sonics). The heart was imaged using M-mode and two-dimensional measurements were made at the level of the papillary muscles. Data represent the average of at least five separate scans in a random-blind fashion. Left ventricular posterior wall thickness at the end-diastolic and end-systolic phases, left ventricular internal diameter in end-diastole and end-systole, and end-diastolic and end-systolic interventricular septum thickness were measured from the M-mode image. FS and EF were used to assess systolic function.

### Masson trichrome staining

Mouse hearts were excised and fixed in 4% paraformaldehyde overnight, embedded in paraffin, and sectioned at 4 μm. Fibrosis was visualized by Masson trichrome staining. Ten fields were chosen randomly and imaged under an Olympus BX51 light microscope at ×20 magnification. ImageJ was used for fibrosis quantification.

### Transmission electron microscopy

Freshly excised hearts were perfused with 1% glutaraldehyde and 4% paraformaldehyde for 30 min. After dissecting into 1–2 mm^3^ blocks, samples were immediately fixed with 2.5% glutaraldehyde and 4% paraformaldehyde, then post-fixed with 1% osmium tetroxide. After dehydration in a graded series of acetone, the samples were embedded in Spurr resin and sectioned with a glass knife on a Leica Ultracut R cutter. Ultra-thin sections (70–90 nm) were imaged with a transmission electron microscope (Tecnai G2 20 Twin, FEI). Mitochondrial area was calculated by analyzing images of mitochondria with a clear outer membrane on the micrographs using ImageJ freehand selection, and the number of pixels was converted to the actual mitochondrial cross-sectional area according to the magnification and pixel size of each micrograph. Mitochondrial volume fraction was analyzed by point-counting method.^58^ Briefly, a square lattice grid of 400 intersections was placed over each micrograph and the number of times a mitochondrion located at an intersection was counted. The final volume fraction was calculated by dividing the number with the total intersection number.

### Isolation of adult mouse cardiomyocytes

Single ventricular myocytes were enzymatically isolated from the hearts of WT, *Ndufab1* cardiac-specific knockout, or pan-tissue transgenic mice, as described previously.^59,60^ Freshly isolated cardiomyocytes were plated on laminin-coated (Sigma) culture dishes for 1 h and then the attached cells were maintained in Dulbecco’s modified Eagle’s medium (Invitrogen, Carlsbad, CA, USA) along with 10% FBS (Hyclone), 5 mM BDM (Sigma), and 1% insulin-transferrin-selenium supplement (Invitrogen) until use.

### Isolation and culture of neonatal cardiomyocytes

Ventricular myocytes were isolated from 1-day-old Sprague-Dawley rats, as described previously.^61^ Myocytes were plated at 1.5 × 10^5^ cells/cm^2^ in DMEM (Invitrogen) supplemented with 10% FBS (Gibco) in the presence of 0.1 mM 5-bromo-2-deoxyuridine (Sigma). Adenovirus infection or siRNA transfection was implemented after 24 h quiescence in serum-free DMEM following 48–72 h culture in DMEM containing 10% FBS. For adenovirus infection, cells were infected with adenovirus carrying LacZ or the *Ndufab1* gene at an m.o.i. of 20. For siRNA transfection, 100 nM siRNA was transiently transfected using Lipofectamine RNAiMax (Invitrogen) according to the manufacturer’s instructions. The knockdown efficiency was assessed by western blot. The double-stranded RNA sequences for knockdown of rat *Ndufab1* gene and the negative control (NC) are listed in the following table:

**Table:** 

siRNA name	Sense	Anti-sense
siNdufab1-1	CACUGACGUUAGAAGGAAUTT	AUUCCUUCUAACGUCAGUGTT
siNdufab1-2	GCUCUCAGUAAAUUCUCAUTT	AUGAGAAUUUACUGAGAGCTT
NC	UUCUCCGAACGUGUCACGUTT	ACGUGACACGUUCGGAGAATT

### Isolation and respiration measurement of cardiac mitochondria

Mouse hearts were washed with ice-cold isolation buffer (210 mM mannitol, 70 mM sucrose, 5 mM HEPES (pH 7.4), 1 mM EGTA, and 1 mg/mL BSA), minced, and homogenized. After the homogenate was centrifuged at 4 °C for 10 min at 700 × *g*, the supernatant was collected and further centrifuged at 4 °C for 10 min at 12000 × *g*. The pellet was re-suspended for functional assessment. The protein concentration of the mitochondrial preparation was determined with a NanoDrop Microvolume Spectrophotometer.

Mitochondrial respiration was evaluated by measuring oxygen consumption with a Clark-type oxygen electrode (Strathkelvin 782 2-Channel Oxygen System version 1.0; Strathkelvin Instruments, Motherwell, UK). Briefly, isolated mitochondria were re-suspended in respiration buffer (in mM: 225 mannitol, 75 sucrose, 10 KCl, 10 Tris-HCl, 5 KH_2_PO_4_, pH 7.2) at 25 °C with different substrates. State II and III OCRs were each measured in the absence and presence of 100 μM ADP. The respiratory control ratio was defined as the ratio of the state III to the state II respiratory rate.

### BN-PAGE analysis of mitochondrial SCs and in-gel activity

BN-PAGE was conducted using the NativePAGE^TM^ system (Invitrogen). Briefly, isolated cardiac mitochondria were solubilized by digitonin (4 g/g protein) for 15 min on ice, then centrifuged at 15,000 rpm at 4 °C for 30 min. After centrifugation, the supernatants were collected and the protein concentration was determined by BCA analysis (ThermoFisher). Coomassie blue G-250 (Invitrogen) was added to the supernatant to obtain a dye/detergent mass ratio of 4/1 and then the protein was loaded into a 4–16% non-denaturing polyacrylamide gel (Invitrogen). After electrophoresis, proteins were transferred to a PVDF membrane and then probed with specific antibodies against subunits of complex I (NDUFB8), complex II (SDHA), complex III (UQCRFS1 and UQCRC1), complex IV (COX IV), and complex V (ATPB or ATP5A). Blots were visualized using secondary antibodies conjugated with IRDye (LI-COR, Lincoln, NE, USA) and an Odyssey imaging system (LI-COR). The anti-NDUFB8, anti-UQCRFS1, or anti-COXIV immunoblot bands with high molecular weight were used to indicate SCs containing complex I, complex III, or complex IV.

For in-gel activity analysis, 2.5 mg/mL nitro blue tetrazolium and 0.5 mg/mL NADH in 2 mM Tris-HCl (pH 7.4) were added to the 4–16% non-denaturing polyacrylamide gel after electrophoresis and incubated for 15 min at 37 °C. The reaction was stopped with 10% acetic acid. The activity was determined by analyzing the color development using the Odyssey imaging system (LI-COR).

### Whole-cell respiration analysis

Mitochondrial respiration was measured using the Seahorse XF24 Extracellular Flux Analyzer (Seahorse Bioscience, North Billerica, MA, USA) according to the manufacturer’s instructions. Briefly, isolated cardiomyocytes were seeded onto an XF24 microplate at 300 cells/well. The cellular OCR was monitored in unbuffered assay medium (Sigma D5030) supplemented with (in mM) 2 GlutaMAX (Gibco), 2.5 sodium pyruvate, and 25 glucose (pH 7.4 at 37 °C), following the sequential addition of oligomycin (1 μM), carbonyl cyanide 4-(trifluoromethoxy) phenylhydrazone (FCCP, 500 nM), and rotenone (1 μM) and antimycin A (1 μM) or in KHB buffer (in mM, 111.3 NaCl, 4.7 KCl, 2.0 MgSO_4_, 1.2 Na_2_HPO_4_, 2.5 glucose, 0.5 L-Carnitine). For fatty acid supported respiration, the 2.5 mM sodium pyruvate and 25 mM glucose were replaced with 200 μM palmitate. Maximal OCR was calculated by subtracting the OCR in the presence of rotenone and antimycin A from that in the presence of FCCP.

### Electron flow assay

The electron flow in isolated mitochondria was measured using the Seahorse XF24 Extracellular Flux Analyzer (Seahorse Bioscience, North Billerica, MA, USA) according to the manufacturer’s instructions. Briefly, 2.5 μg of mouse heart mitochondria were plated in each well of the XF24 plate containing 1 × MAS (in mM: 70 sucrose, 220 mannitol, 5 KH_2_PO_4_, 5 MgCl_2_, 2 HEPES, 1 EGTA, 0.2% BSA, pH 7.4 at 37 °C) supplemented with 10 mM pyruvate, 2 mM malate and 4 μM FCCP. The mitochondrial OCR was monitored following the sequential addition of rotenone (2 μM), succinate (10 mM), antimycin A (4 μM) and ascorbate/TMPD (10 mM/100 μM). The electron flow of different complexes was revealed by their inhibitor-sensitive OCR.

### Measurement of H_2_O_2_ production

H_2_O_2_ production in isolated mitochondria was measured using the Amplex® Red hydrogen peroxide kit (Molecular Probes). Briefly, isolated mitochondria were incubated in an assay medium with 10 mM succinate, 50 mM Amplex red, and 5 units/mL HRP in each well of a 96-well plate. The increase in Amplex red fluorescence was followed over 60 min at room temperature with excitation at 530 nm and emission at >590 nm in a 96-well microplate reader (Biotek, Winooski, VT, USA). H_2_O_2_ production was indexed by the increase of Amplex red fluorescence per min.

### ATP measurement in isolated cardiomyocytes

The ATP was extracted from fresh isolated mouse cardiomyocytes with cold 2.5% trichloroacetic acid. The cellular ATP content was measured by luciferase assay according to manufacturer’s instructions (Promega, Madison, WI, USA).

### NAD^+^, NADH, and cardiolipin measurement

For NAD^+^ and NADH measurement, 20 mg heart tissue was homogenized and measured with a commercially available kit according to the manufacturer’s instructions (BioAssay). For cardiolipin measurement, 100 μg mitochondria were lysed by freeze thawing and measured with a commercially available kit according to the manufacturer’s instructions (BioVision).

### In vivo and ex vivo myocardial IR models

For in vivo IR surgery, 2–4-month-old male mice were anesthetized with avertin (2.5%, 0.01 mL/g, i.p.) and ventilated via a tracheostomy on a Minivent respirator. A midline sternotomy was performed, and a reversible coronary artery snare occluder was placed around the left anterior descending coronary artery. Myocardial IR was induced by tightening the snare for 30 min and then loosening it for 24 h. To measure the infarct size, the animals were anesthetized with avertin (2.5%, 0.015 mL/g, i.p.) and heparinized (400 USP U/kg, i.p.) at the end-point. The heart was excised and the ascending aorta was cannulated (distal to the sinus of Valsalva), then perfused retrogradely with Alcian blue dye (1%) to visualize the area not at risk. The coronary artery was re-occluded at the site of occlusion before perfusion with Alcian blue. Then, the heart was frozen at −80 °C for 10 min and cut into 1 mm slices (5–6 slices per heart), which were then incubated in sodium phosphate buffer containing 1.5% 2,3,5-triphenyl-tetrazolium chloride for 15 min to visualize the unstained infarcted region. Infarct area, area at risk (AAR), and left ventricular area were determined by planimetry using ImageJ. The infarct size was calculated as infarct area divided by AAR. For LDH measurement, blood samples were collected before surgery and after 24 h of reperfusion, and centrifuged for 10 min at 3000 rpm to obtain serum. LDH was spectrophotometrically assayed using a kit from Jingyuan Biology (Shanghai, China).

For ex vivo IR, the heart was excised from male mice (2–4 months old), and the ascending aorta was cannulated with a customized needle. The heart was perfused in the Langendorff configuration under constant perfusion pressure (1000 mm H_2_O) with oxygenated (100% O_2_) Tyrode’s solution (pH 7.4 at 37 °C). The Langendorff-perfused heart was placed on the stage of the confocal microscope and images were captured ~30 μm deep into the epimyocardium of the left ventricle. Motion artifacts due to spontaneous beating of the heart (~480/min) were minimized using 10 µM blebbistatin (Sigma). After a 10-min stabilization period, the heart was perfused with 15 μM DCF (an indicator of ROS) or 500 nM TMRM (an indicator of mitochondrial membrane potential) and 0.05% EBD (an indicator of plasma membrane integrity) for 20 min. The IR protocol used was as described previously.^51,52^ Ischemia (30 min) was achieved by clamping the perfusion line and reperfusion (30 min) by releasing the clamp. Confocal images were captured from multiple, randomly selected regions prior to, during ischemia, and after the IR, at excitation wavelengths of 488, 543, and 633 nm and emission wavelengths of 500–540, 565–595, and >650 nm for DCF, TMRM, and EBD, respectively.

### Confocal microscopy

An inverted confocal microscope (Zeiss LSM 710) with a 40×, 1.3 NA oil-immersion objective was used for imaging. For mitoSOX measurement, the indicator (5 μM) was loaded into isolated cardiomyocytes at 37 °C for 30 min followed by three washes with Tyrode’s solution. The mitoSOX fluorescence was measured by excitation at 514 nm and emission at 580–740 nm. To measure mitochondrial membrane potential in isolated cardiomyocytes, TMRM (50 nM) was loaded at 37 °C for 10 min followed by three washes and the fluorescence was measured by excitation at 543 nm and emission at >560 nm. The intensity of FCCP (5 μM)-sensitive TMRM fluorescence was calculated and used to assess mitochondrial membrane potential.

### RNA-seq analysis

Using TRIzol reagent (Invitrogen), total RNA was isolated from left ventricular myocardium in cKO and WT male mice (8–16 weeks old) according to the manufacture’s protocol. The quality of the extracted RNA was controlled using Agilent 2100. A sequencing library was prepared using the NEBNext®Ultra^TM^ RNA Library Prep Kit for Illumina® (New England Biolabs, Ipswich, MA, USA) following the manufacturer’s recommendations. Deep sequencing was then performed on an Illumina Hiseq 4000 platform and 150 bp paired-end reads were generated. To obtain the differentially expressed genes, RNA-seq reads were first aligned to the mouse genome (genome version mm9) with tophat2 (version 2.1.1; main parameters: –read-mismatches 8, –min-anchor 8, –segment-length 30, –segment-mismatches 2) and all the mapping results were evaluated as previously described.^62^ DEseq2 packages were used to obtain differentially expressed genes with a cutoff of fold-change in parame *P*-value < 0.05. The functional annotation and pathway enrichment analysis for both upregulated and downregulated genes were performed using Database for Annotation, Visualization, and Integrated Discovery^63,64^ with a *P*-value cutoff of 0.05 under the Benjamini test.

### Western blot analysis

Isolated cardiomyocytes were homogenized with denaturing lysis buffer, and 10–50 μg of protein per sample was separated on 12% SDS-PAGE. After electrophoresis, proteins were transferred to a PVDF membrane and then probed with specific antibodies. Blots were visualized using secondary antibodies conjugated with IRDye (LI-COR) and an Odyssey imaging system (LI-COR). The antibodies for NDUFAB1, SDHB, and SDHD were from Origene, those for NDUFB8, NDUFS4, SDHA, UQCRC1, UQCRB, ATPB, ATP5A, AcK103, and lipoic acid from Abcam, and those for NDUFS1, NDUFS6, NDUFS7, UQCRFS1, COXIV, ISCU, and NFS1 from ProteinTech.

### RNA isolation and real-time PCR (RT-PCR)

Total RNA was isolated from freshly isolated mouse cardiomyocytes with TRIzol reagent (Invitrogen) according to the manufacturer’s instructions, and then converted to cDNA using TransScript One-Step gDNA Removal and cDNA Synthesis Mix (Transgen Biotech). Quantitative RT-PCR reactions were performed using Trans Start Green qPCR Super Mix (TransGen Biotech) and a BioRad CFX96 Touch RT-PCR Detection System. The primers used were as follows:

**Table:** 

**Gene**	**Forward**	**Reverse**
Ndufab1	CTGAGGGAATCCGGAGGAGA	GTCTAACGTCAGTGGGGGTG
Ndufs1	CTCCTCTTGCCCTTGACTGG	CCAGCCCTTCATTACAGGCA
Ndufs4	ATCCACTTGGAAGCTGGCAGACAA	AGACTGCATGTTATTGCGAGCAGG
Ndufs6	CCTGGAGCGATTCTGGGATAACTT	TGGGCTTCGAGCTAACAATGGTGT
Ndufs7	CAGAGTTCATCAGAGTGTAGCC	CGGGGAAGATGAGAGAGCTTG
Ndufb8	CATCTCTTCGGCTTTGTGGC	CAAAAAGCCCATCAAGCCTCC
Sdha	AGATTGTGCCTGGTCTGTATGCCT	AGGCTCTGCCAAAGACTACAAGGT
Sdhb	ACCCCTTCTCTGTCTACCG	AATGCTCGCTTCTCCTTGTAG
Sdhd	TGGTACCCAGCACATTCACC	GGGTGTCCCCATGAACGTAG
Uqcrc1	GACAACGTGACCCTCCAAGT	AGGATGTTTTTGCCCCGAGT
Uqcrc2	GGCTTGTTCGTTAAAGCAGGCAGT	TGCCTTCTACAGTGTACGCCATGT
Uqcrfs1	TCCCTGAAGGGAAGAACATGGCT	TGCAGCTTCCTGGTCAATCTCCTT
Gapdh	ACCACAGTCCATGCCATCAC	TCCACCCACCCTGTTGCTGTA

### Statistics

Data are expressed as mean ± s.e.m. When appropriate, Student’s *t*-test was applied to determine statistical significance. The log-rank test was used for survival curves. *P* < 0.05 was considered statistically significant.

## Supplementary information

Supplementary information Fig. S1

Supplementary information Fig. S2

Supplementary information Fig. S3

Supplementary information Fig. S4

Supplementary information Fig. S5

Supplementary information Fig. S6

Supplementary information Fig. S7

Supplementary information Fig. S8

Supplementary information Fig. S9

Supplementary information Fig. S10

Supplementary information Fig. S11

Supplementary information Fig. S12

Supplementary information Fig. S13

Supplementary information Fig. S14

Supplementary information Fig. S15

Supplementary information Fig. S16

Supplementary information Fig. S17

Supplementary information Fig. S18

Supplementary information Fig. S19

Supplementary information Table S1

Supplementary information Table S2

Supplementary information Table S3

Supplementary information Table S4
