# Supplementary material for: NDUFAB1 confers cardio-protection by enhancing mitochondrial bioenergetics through coordination of respiratory complex and supercomplex assembly
Source: Cell Res. 2019 Jul 31;29(9):754–66. doi: 10.1038/s41422-019-0208-x (PMC6796901; doi:10.1038/s41422-019-0208-x)
Supplement: Supplementary file 1 — Supplementary information Fig. S1 [file 41422_2019_208_MOESM1_ESM.pdf]

Fig. S1

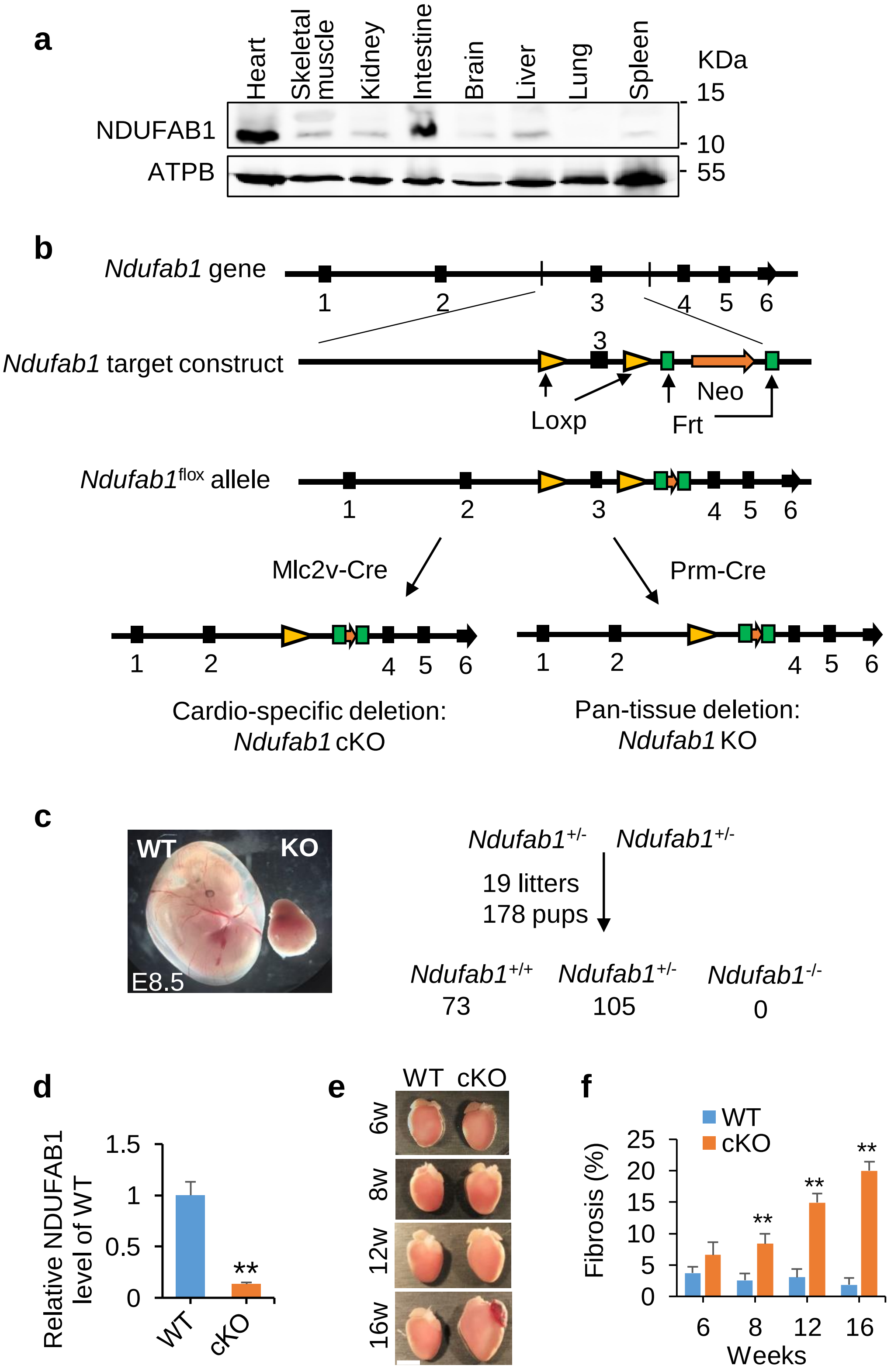

a
Heart
Skeletal muscle
Kidney
Intestine
Brain
Liver
Lung
Spleen
KDa
15
10
55
NDUFAB1
ATPB
b
Ndufab1 gene
1
2
3
4
5
6
Ndufab1 target construct
3
Neo
Loxp
Frt
Ndufab1flox allele
Mlc2v-Cre
Prm-Cre
Cardio-specific deletion:
Ndufab1 cKO
Pan-tissue deletion:
Ndufab1 KO
c
WT
KO
E8.5
Ndufab1+/-
Ndufab1+/-
19 litters
178 pups
Ndufab1+/+
73
Ndufab1+/-
105
Ndufab1-/-
0
d
Relative NDUFAB1 level of WT
1.5
1
0.5
0
**
WT
cKO
e
WT cKO
6w
8w
12w
16w
f
WT
cKO
Fibrosis (%)
25
20
15
10
5
0
**
6
8
12
16
Weeks

Fig. S1. Generation and phenotyping of *Ndufab1* cardiac-specific knockout (cKO) or pan-tissue knockout (KO) mouse models.

(a) Western blots for *Ndufab1* expression in different mouse organs. ATPB served as the loading control.

(b) Schematic of gene-targeting strategy. LoxP sites flank exon 3 of the *Ndufab1* gene and Frt sites flank the neomycin resistance (Neo) cassette. *Ndufab1*$^{flox/flox}$ mice were crossed with Mlc2v-Cre or Prm-Cre mice to allow cardiomyocyte-specific or pan-tissue deletion of *Ndufab1*, respectively.

(c) Embryonic lethality of pan-tissue knockout of *Ndufab1*. Left, representative images of E8.5 wild-type (WT) and KO embryos. Right, summary of live pups recovered from Ndufab1$^{+/-}$ intercrossing.

(d) Relative expression of NDUFAB1 in WT (flox/flox, Mlc2v-Cre^{-}) and cKO (flox/flox, Mlc2v-Cre^{+}) hearts (mean ± s.e.m.; n = 4 mice per group; ** p <0.01 *versus* WT).

(e) Representative images of gross heart morphology at 6-, 8-, 12-, and 16-weeks (w) of age. Note that a blood clot has pooled in the left atrial appendage of the 16-week old cKO heart (scale bar, 5 mm).

(f) Statistics of fibrosis (as shown in Figure 1g) (mean ± s.e.m.; n = 20–45 fields from 3 hearts per group; ** p <0.01 *versus* WT).
