# Supplementary material for: NDUFAB1 confers cardio-protection by enhancing mitochondrial bioenergetics through coordination of respiratory complex and supercomplex assembly
Source: Cell Res. 2019 Jul 31;29(9):754–66. doi: 10.1038/s41422-019-0208-x (PMC6796901; doi:10.1038/s41422-019-0208-x)
Supplement: Supplementary file 2 — Supplementary information Fig. S2 [file 41422_2019_208_MOESM2_ESM.pdf]

Fig. S2

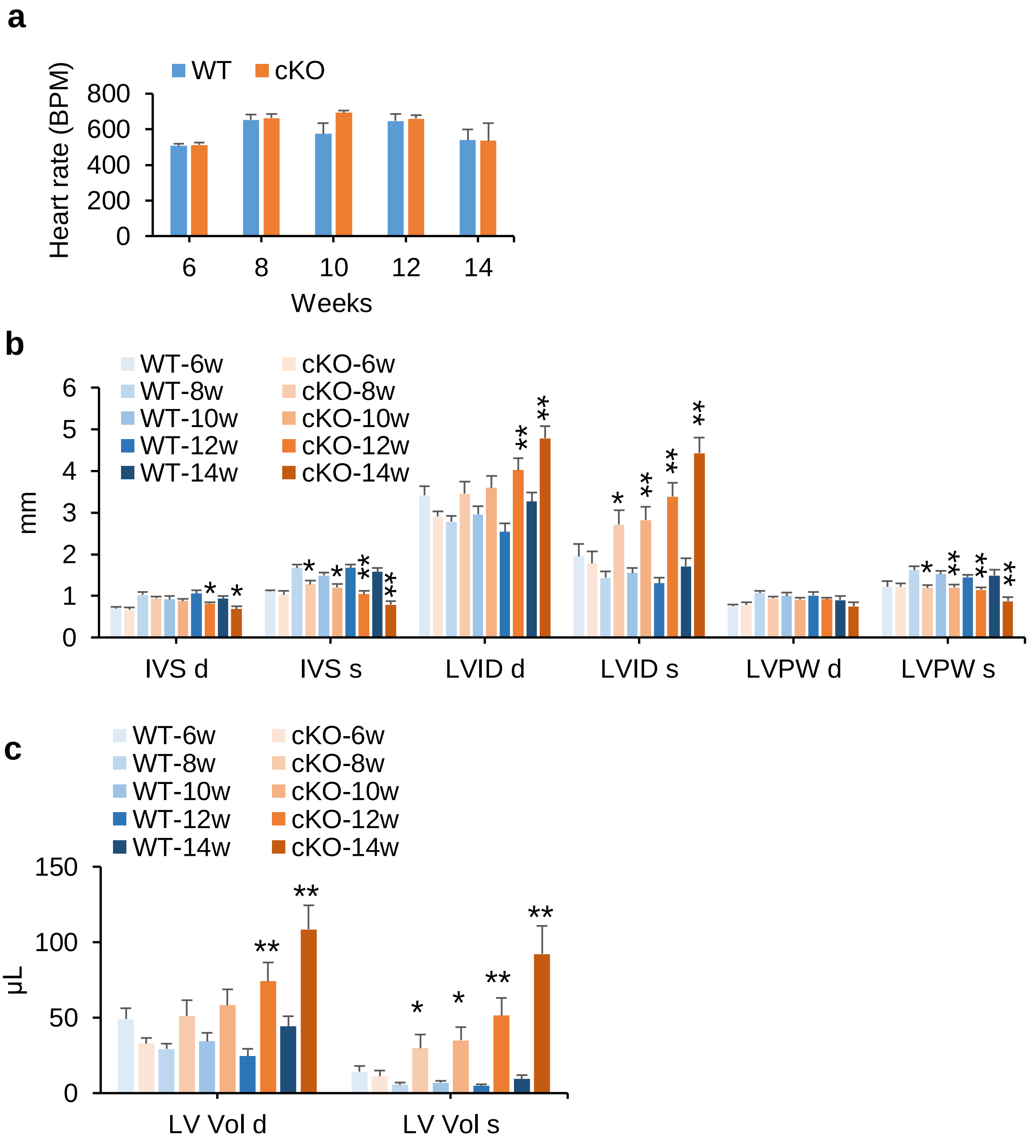


**Fig. S2. Echocardiographic analysis of cardiac functions of WT and cKO mice at different ages.**

**(a)** Heart rate (BPM, beats per minute; mean ± s.e.m.; n = 3–10 mice per group).
**(b)** Interventricular septum thickness at end-diastole and end-systole (IVS d, s), left ventricular internal diameter at end-diastole and end-systole (LVID d, s), and left ventricular posterior wall thickness at end-diastole and end-systole (LVPW d, s) (mean ± s.e.m.; n = 3–9 mice per group; * p <0.05, ** p <0.01 *versus* WT).
**(c)** Left ventricular volume at end-diastole and end-systole (LV Vol d, s) (mean ± s.e.m.; n = 3–9 mice per group; * p <0.05, ** p <0.01 *versus* WT).
