# Supplementary material for: NDUFAB1 confers cardio-protection by enhancing mitochondrial bioenergetics through coordination of respiratory complex and supercomplex assembly
Source: Cell Res. 2019 Jul 31;29(9):754–66. doi: 10.1038/s41422-019-0208-x (PMC6796901; doi:10.1038/s41422-019-0208-x)
Supplement: Supplementary file 3 — Supplementary information Fig. S3 [file 41422_2019_208_MOESM3_ESM.pdf]

Fig. S3

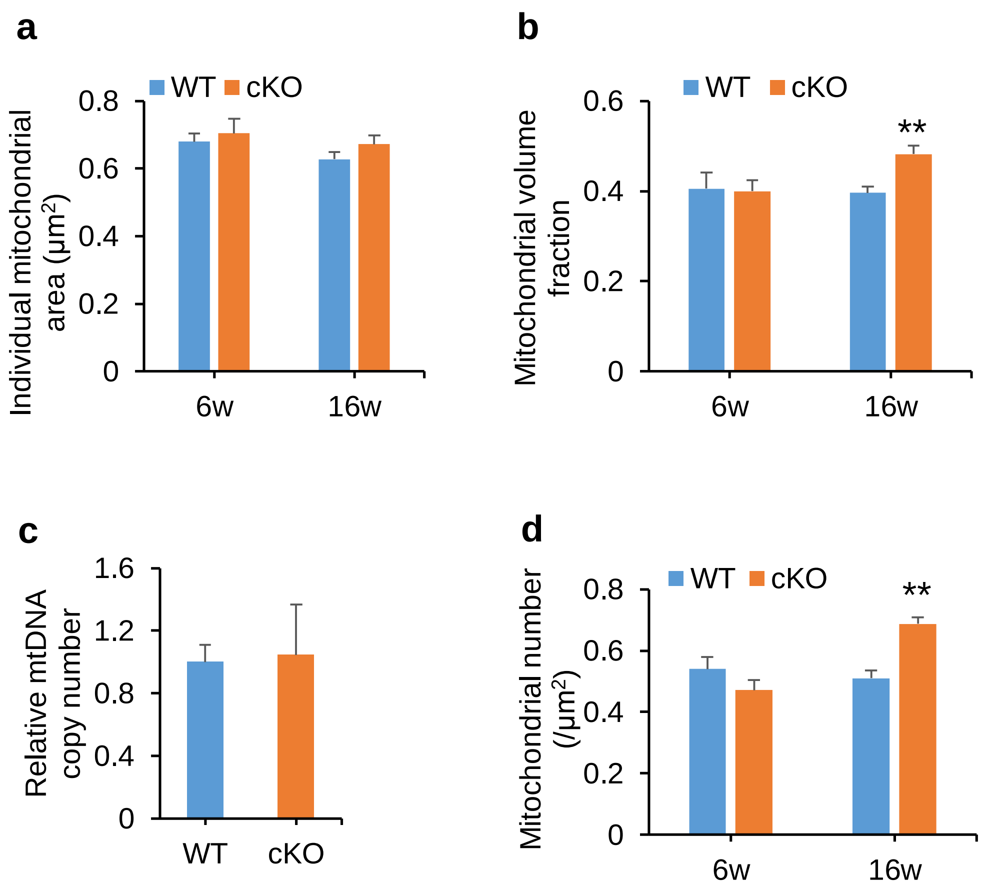

Fig. S3. Individual mitochondrial area **(a),** total volume fraction **(b)**, and mitochondrial number **(d)** quantified from electron micrographs at the age of 6 and 16 weeks (as shown in Figure 2a) (mean ± s.e.m.; n = 85–172 mitochondria for **(a)**, 11–39 images for **(b)**, and 9-16 images for **(d)**; 3 mice per group; ** $p < 0.01$ *versus* WT). **(c)** Mitochondrial DNA content in WT and cKO mice heart (mean ± s.e.m.; n = 5-6 mice).
