# Supplementary material for: NDUFAB1 confers cardio-protection by enhancing mitochondrial bioenergetics through coordination of respiratory complex and supercomplex assembly
Source: Cell Res. 2019 Jul 31;29(9):754–66. doi: 10.1038/s41422-019-0208-x (PMC6796901; doi:10.1038/s41422-019-0208-x)
Supplement: Supplementary file 4 — Supplementary information Fig. S4 [file 41422_2019_208_MOESM4_ESM.pdf]

Fig. S4

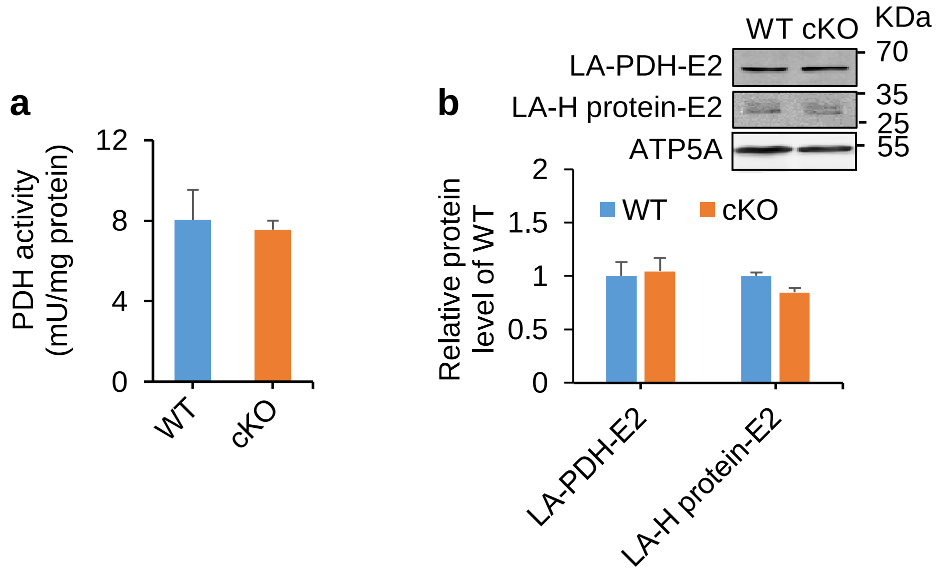

Fig. S4. Little effect of NDUFAB1 ablation on pyruvate dehydrogenase (PDH) activity and protein lipoylation status.

(a) PDH activity in WT and cKO mitochondria (mean ± s.e.m.. n = 3-4 mice per group).

(b) Western blots of lipoic acid (LA)-conjugated E2 of PDH complex and H protein. ATP5A served as the loading control. Data are mean ± s.e.m.. n = 3-6 mice per group.
