# Supplementary material for: NDUFAB1 confers cardio-protection by enhancing mitochondrial bioenergetics through coordination of respiratory complex and supercomplex assembly
Source: Cell Res. 2019 Jul 31;29(9):754–66. doi: 10.1038/s41422-019-0208-x (PMC6796901; doi:10.1038/s41422-019-0208-x)
Supplement: Supplementary file 5 — Supplementary information Fig. S5 [file 41422_2019_208_MOESM5_ESM.pdf]

Fig. S5

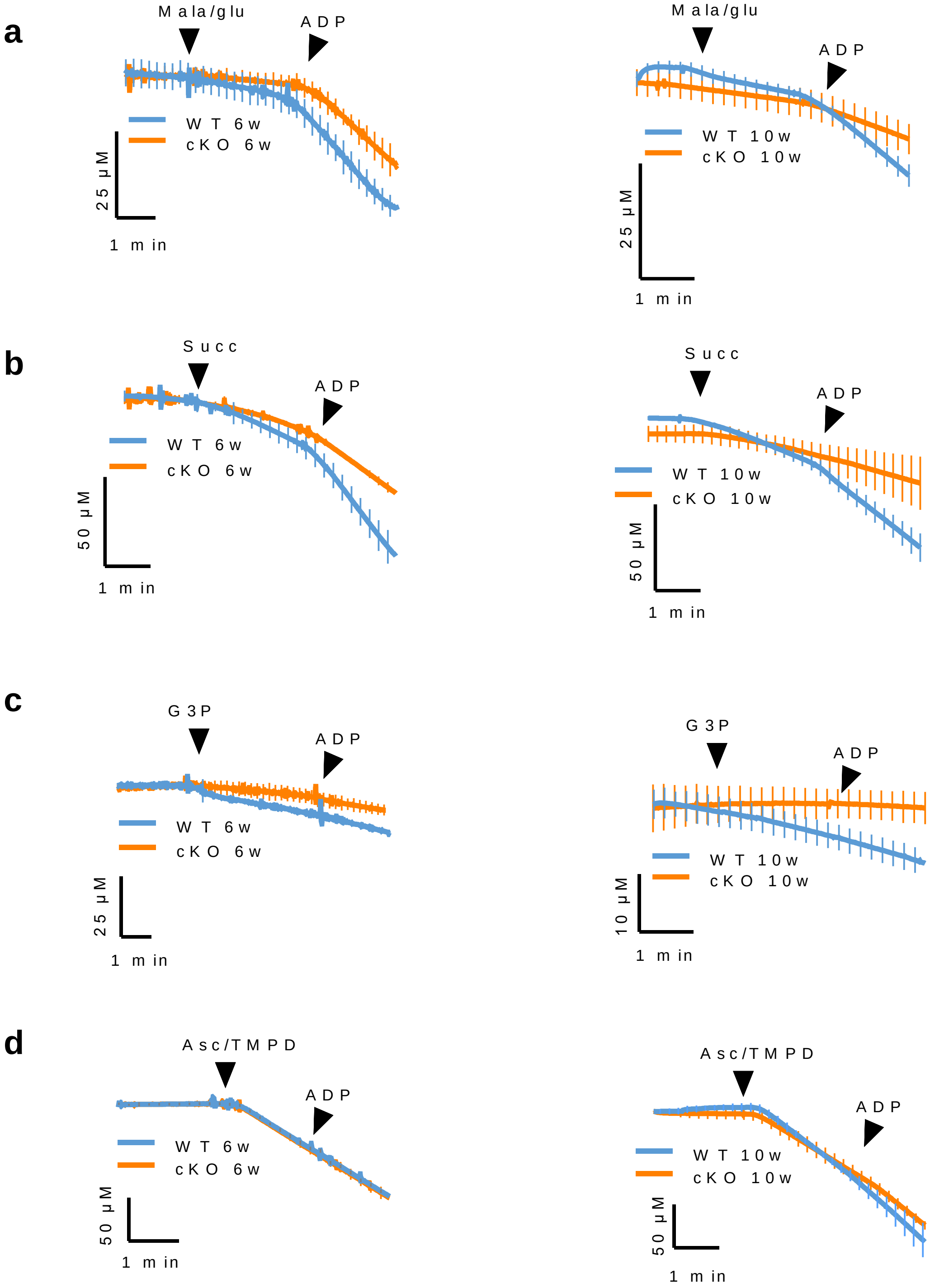

Fig. S5. Average traces of oxygen consumption recorded with Clark electrode in 6-week and 10-week cKO and WT mitochondria in presence of malate/glutamate for complex I **(a)**, succinate for complex II **(b)**, glycerol-3-phosphate for complex III **(c)** and ascorbate/TMPD for complex IV **(d)**. Data are mean ± s.e.m.; n = 3–5 male mice per group.
