# Supplementary material for: NDUFAB1 confers cardio-protection by enhancing mitochondrial bioenergetics through coordination of respiratory complex and supercomplex assembly
Source: Cell Res. 2019 Jul 31;29(9):754–66. doi: 10.1038/s41422-019-0208-x (PMC6796901; doi:10.1038/s41422-019-0208-x)
Supplement: Supplementary file 6 — Supplementary information Fig. S6 [file 41422_2019_208_MOESM6_ESM.pdf]

Fig. S6

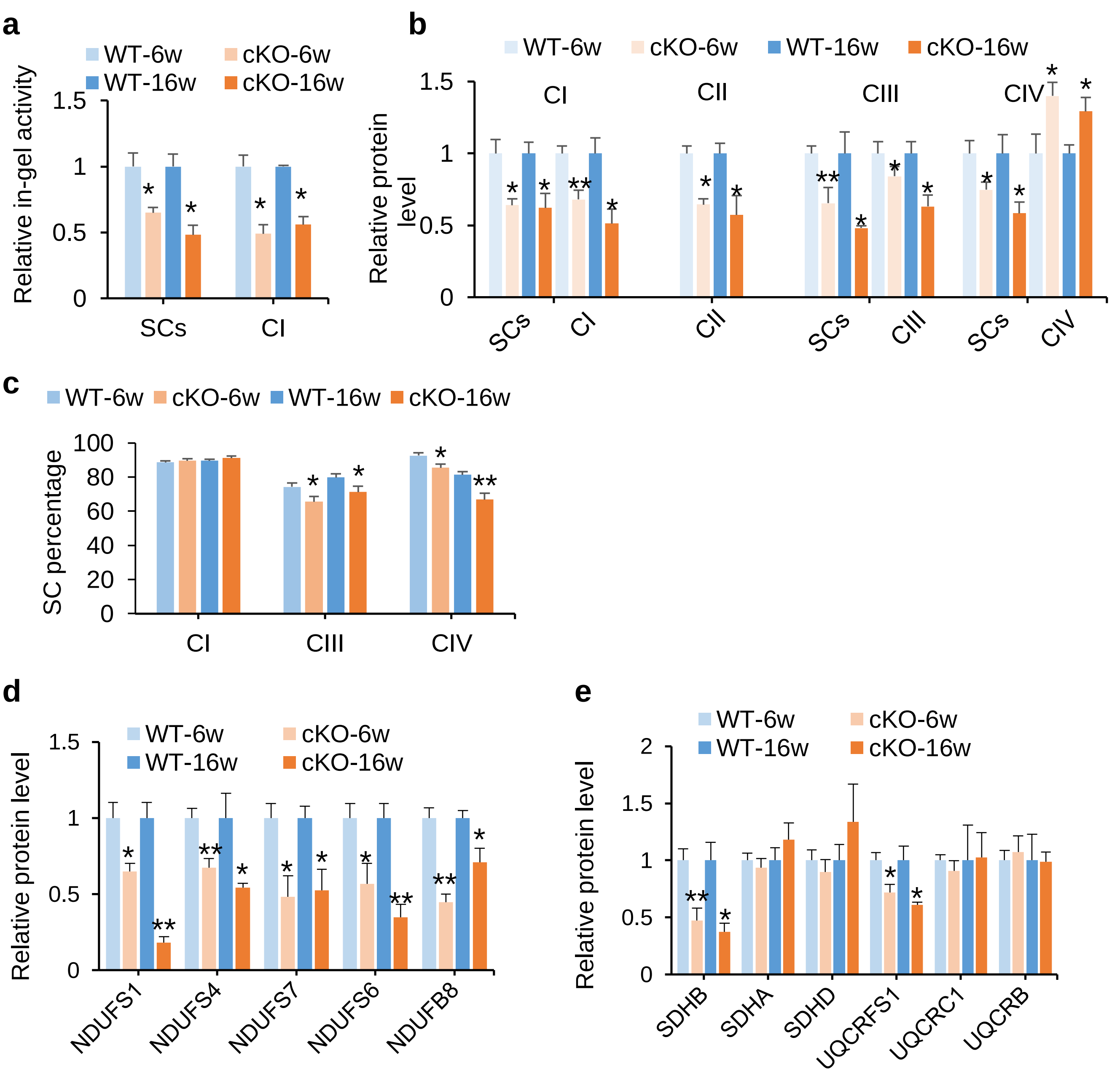

Fig. S6. Statistics of Figure 3B-3F.
(a) Statistics of in-gel activity of SCs and complex I.
(b) Statistics of BN-PAGE immunoblots of individual ETC complexes and SCs.
(c) Percentages of individual ETC complex-containing SCs.
(d) Statistics of expression of complex I subunits.
(e) Statistics of expression of subunits of complex II or III.
Isolated mitochondria or cardiomyocytes from 6- or 16 week-old mice were used. The cKO activity or expression was normalized to WT at the same age (mean ± s.e.m.; n = 3–8 male mice per group; * $p<0.05$, ** $p<0.01$ versus WT). For CIII, anti-UQCRFS1 blots were used.
