# Supplementary material for: NDUFAB1 confers cardio-protection by enhancing mitochondrial bioenergetics through coordination of respiratory complex and supercomplex assembly
Source: Cell Res. 2019 Jul 31;29(9):754–66. doi: 10.1038/s41422-019-0208-x (PMC6796901; doi:10.1038/s41422-019-0208-x)
Supplement: Supplementary file 7 — Supplementary information Fig. S7 [file 41422_2019_208_MOESM7_ESM.pdf]

Fig. S7

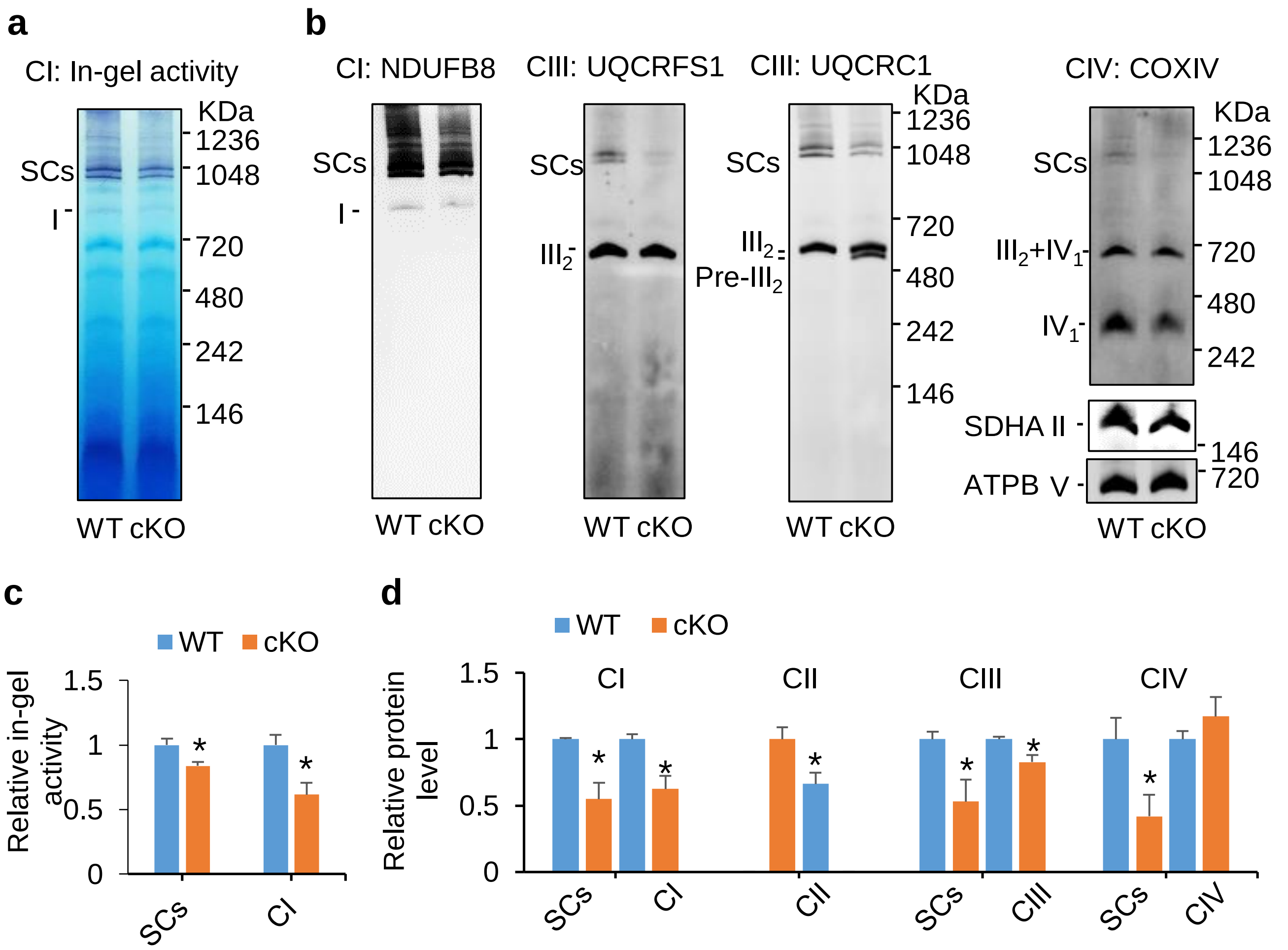

Fig. S7. Impaired assembly of ETC complexes I-III and SCs in the cardiac mitochondria of newborn cKO mice (born within 24 h).

(a) In-gel activity of SCs and complex I in WT and cKO mitochondria from newborn mice.

(b) BN-PAGE immunoblots of individual ETC complexes and SCs. The antibodies used were as the same as Figure 3C.

(c) Statistics of **(a)**.

(d) Statistics of **(b)**.

The cKO activity or expression was normalized to WT (mean ± s.e.m.; n = 3–5 mice per group; * $p<0.05$ versus WT). For CIII, anti-UQCRFS1 blots were used.
