# Supplementary material for: NDUFAB1 confers cardio-protection by enhancing mitochondrial bioenergetics through coordination of respiratory complex and supercomplex assembly
Source: Cell Res. 2019 Jul 31;29(9):754–66. doi: 10.1038/s41422-019-0208-x (PMC6796901; doi:10.1038/s41422-019-0208-x)
Supplement: Supplementary file 8 — Supplementary information Fig. S8 [file 41422_2019_208_MOESM8_ESM.pdf]

Fig. S8

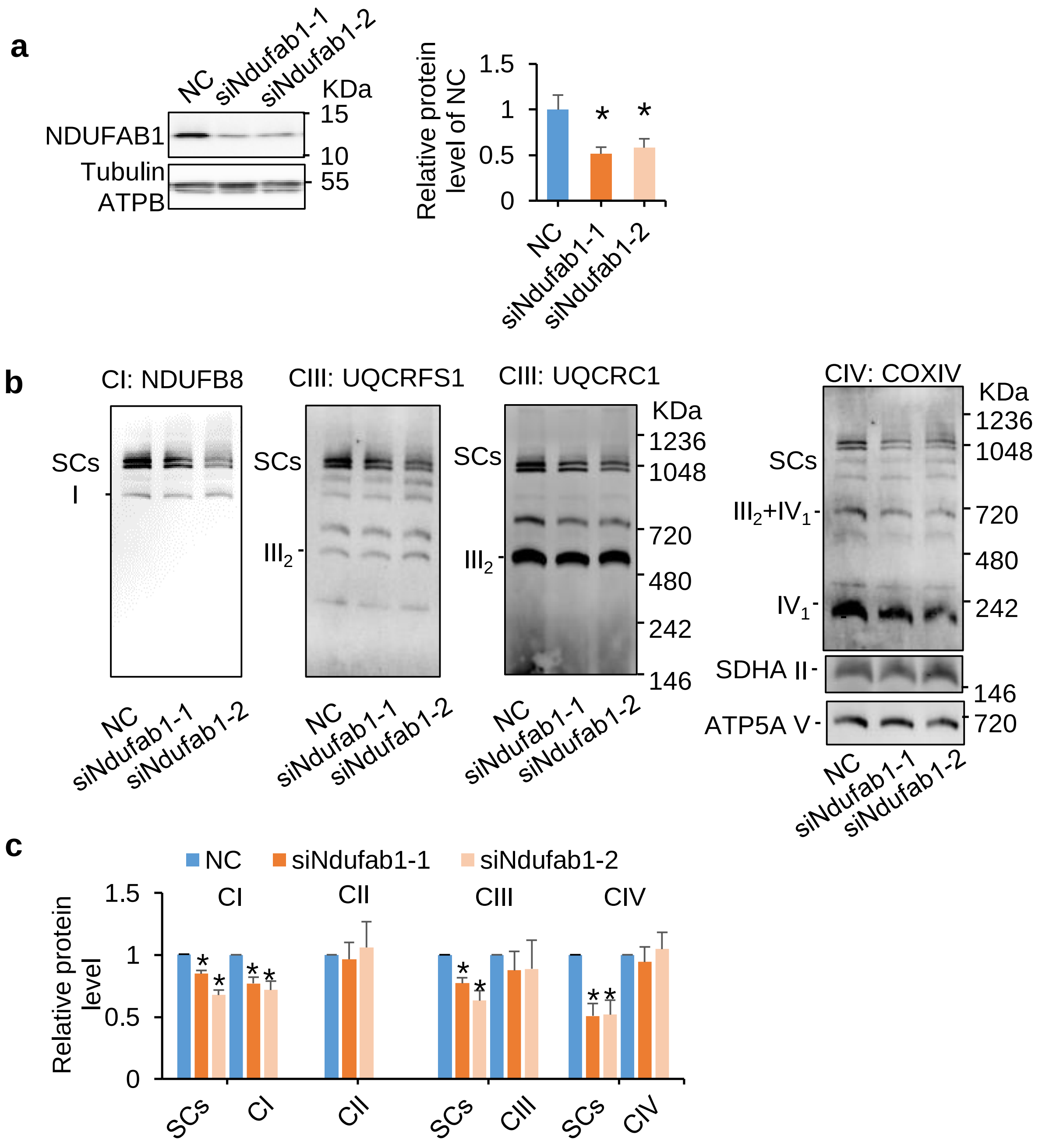


**Fig. S8. Effects of *Ndufab1* knockdown on the assembly of ETC complexes and SCs in cultured neonatal rat cardiomyocytes.**

**(a)** Western blots of NDUFAB1 in the knockdown and control cells. ATPB served as the loading control. siNdufab1-1, siNdufab1-2: two independent siRNAs; NC: negative control siRNA. Data are mean ± s.e.m.; n = 5 per group; * p<0.05 versus NC.

**(b)** BN-PAGE immunoblots of individual ETC complexes and SCs. The antibodies used were as the same as Figure 3C.

**(c)** Statistics of **(b).** The expression was normalized to NC group (mean ± s.e.m.; n = 3–4 per group; * p<0.05 versus NC). For CIII, anti-UQCRFS1 blots were used.
