# Supplementary material for: NDUFAB1 confers cardio-protection by enhancing mitochondrial bioenergetics through coordination of respiratory complex and supercomplex assembly
Source: Cell Res. 2019 Jul 31;29(9):754–66. doi: 10.1038/s41422-019-0208-x (PMC6796901; doi:10.1038/s41422-019-0208-x)
Supplement: Supplementary file 9 — Supplementary information Fig. S9 [file 41422_2019_208_MOESM9_ESM.pdf]

Fig. S9

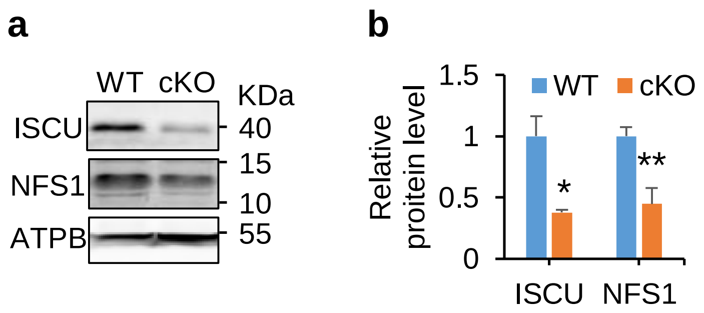

Fig. S9. Decreased protein levels of ISCU and NFS1 in cKO cardiomyocytes.

(a) Representative western blots. ATPB served as the loading control.

(b) Statistics of **(a)** (mean ± s.e.m.; n = 4-7 mice per group; * $p < 0.05$, ** $p < 0.01$ versus WT).
