# Supplementary material for: NDUFAB1 confers cardio-protection by enhancing mitochondrial bioenergetics through coordination of respiratory complex and supercomplex assembly
Source: Cell Res. 2019 Jul 31;29(9):754–66. doi: 10.1038/s41422-019-0208-x (PMC6796901; doi:10.1038/s41422-019-0208-x)
Supplement: Supplementary file 10 — Supplementary information Fig. S10 [file 41422_2019_208_MOESM10_ESM.pdf]

Fig. S10

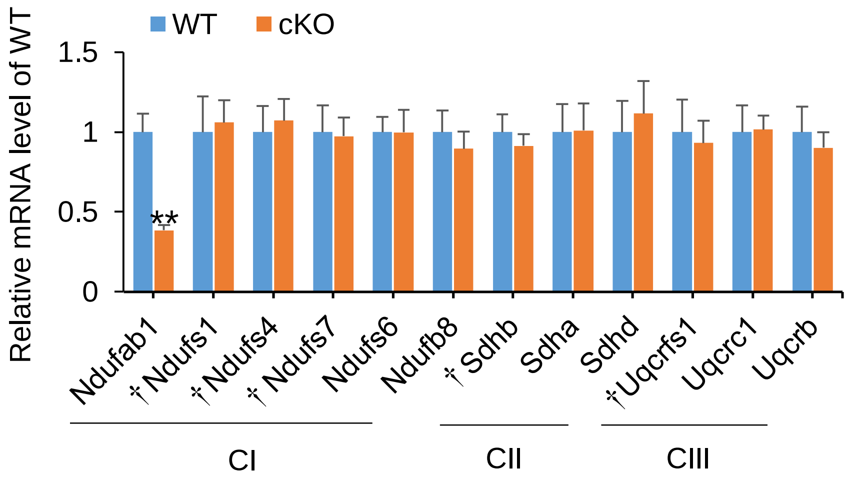

Fig. S10. Real-time PCR analysis of subunit expression of complexes I-III (CI-CIII). The mRNA were extracted from isolated cardiomyocytes. Data are mean ± s.e.m.; n = 4–6 independent experiments per group; ** p <0.01 *versus* WT; †FeS-containing proteins.
