# Supplementary material for: NDUFAB1 confers cardio-protection by enhancing mitochondrial bioenergetics through coordination of respiratory complex and supercomplex assembly
Source: Cell Res. 2019 Jul 31;29(9):754–66. doi: 10.1038/s41422-019-0208-x (PMC6796901; doi:10.1038/s41422-019-0208-x)
Supplement: Supplementary file 11 — Supplementary information Fig. S11 [file 41422_2019_208_MOESM11_ESM.pdf]

Fig. S11

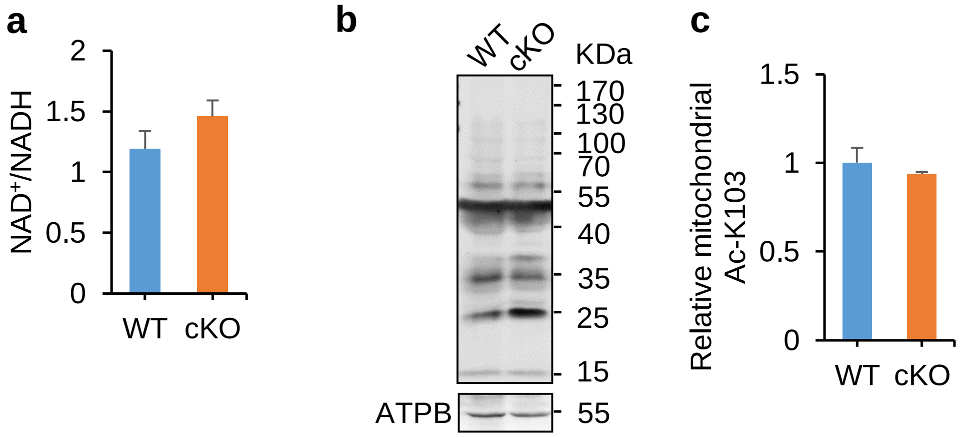

Fig. S11. The NAD$^+$/NADH ratio and protein acetylation in cKO and WT hearts.

(a) NAD$^+$/NADH ratio cKO and WT hearts (mean ± s.e.m.; n = 3 mice per group).

(b) Representative western blots of the acetylome in cKO and WT hearts. ATPB served as the loading control.

(c) Statistics of (**b**) (mean ± s.e.m.; n = 3 mice per group).
