# Supplementary material for: NDUFAB1 confers cardio-protection by enhancing mitochondrial bioenergetics through coordination of respiratory complex and supercomplex assembly
Source: Cell Res. 2019 Jul 31;29(9):754–66. doi: 10.1038/s41422-019-0208-x (PMC6796901; doi:10.1038/s41422-019-0208-x)
Supplement: Supplementary file 12 — Supplementary information Fig. S12 [file 41422_2019_208_MOESM12_ESM.pdf]

Fig. S12

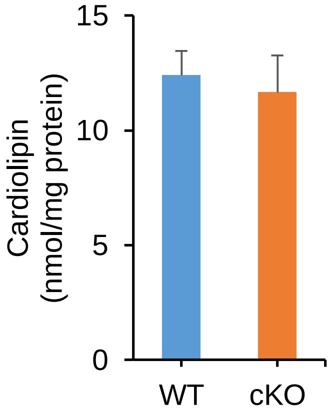

Fig. S12. The mitochondrial cardiolipin content of cKO and WT hearts. The cardiolipin content were normalized by mitochondrial protein amount (mean ± s.e.m.; n = 6 mice per group).
