# Supplementary material for: NDUFAB1 confers cardio-protection by enhancing mitochondrial bioenergetics through coordination of respiratory complex and supercomplex assembly
Source: Cell Res. 2019 Jul 31;29(9):754–66. doi: 10.1038/s41422-019-0208-x (PMC6796901; doi:10.1038/s41422-019-0208-x)
Supplement: Supplementary file 13 — Supplementary information Fig. S13 [file 41422_2019_208_MOESM13_ESM.pdf]

Fig. S13

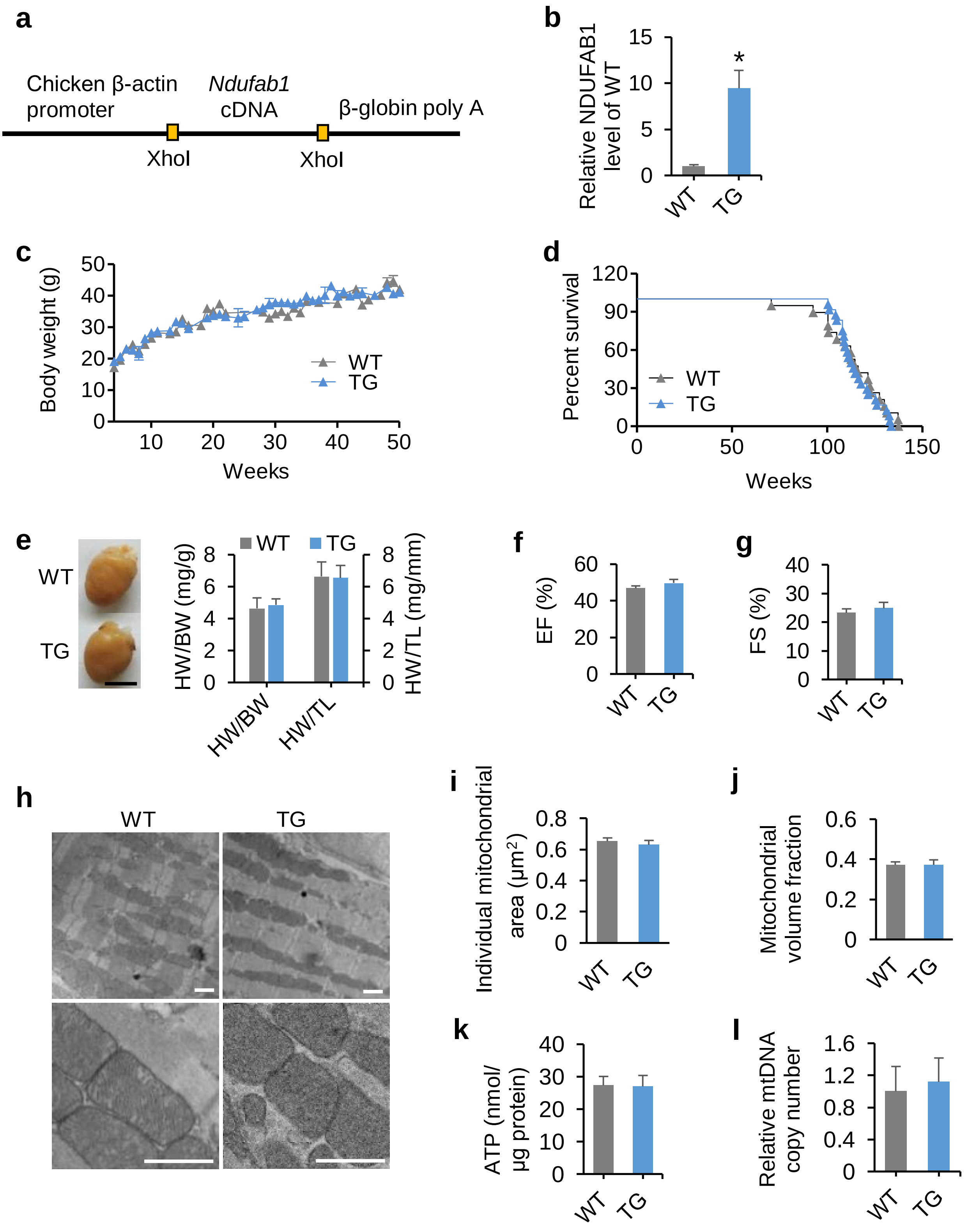
a
Chicken β-actin promoter
Ndufab1 cDNA
β-globin poly A
XhoI
XhoI
b
Relative NDUFAB1 level of WT
15
10
5
0
*
WT
TG
c
Body weight (g)
50
40
30
20
10
0
10
20
30
40
50
Weeks
WT
TG
d
Percent survival
120
90
60
30
0
0
50
100
150
Weeks
WT
TG
e
WT
TG
HW/BW (mg/g)
HW/TL (mg/mm)
8
6
4
2
0
HW/BW
HW/TL
f
EF (%)
60
40
20
0
WT
TG
g
FS (%)
40
30
20
10
0
WT
TG
h
WT
TG
i
Individual mitochondrial area (μm²)
0.8
0.6
0.4
0.2
0
WT
TG
j
Mitochondrial volume fraction
0.6
0.4
0.2
0
WT
TG
k
ATP (nmol/ μg protein)
40
30
20
10
0
WT
TG
l
Relative mtDNA copy number
1.6
1.2
0.8
0.4
0
WT
TG

Fig. S13. Generation and phenotyping of *Ndufab1* transgenic (TG) mice.
(a) Schematic of transgenic construct for generating TG mice. Mouse *Ndufab1* cDNA was cloned into the pUCCAGGS vector downstream of the chicken β-actin promoter.
(b) NDUFAB1 in WT and TG hearts (mean ± s.e.m.; n = 3 mice per group; * $p < 0.05$ *versus* WT).
(c) Growth curves of WT and TG mice (n = 3–15 mice per time point).
(d) Kaplan-Meier survival curves of WT and TG mice (n = 19 for WT and 24 for TG mice).
(e) Cardiac morphology. Left panel, representative photographs of WT and TG hearts (scale bar, 5 mm). Right panel, ratios of heart weight (HW) to body weight (BW) or tibial length (TL) (mean ± s.e.m.; n = 7–36 mice per group). The mice were 12 weeks old.
(f, g) Echocardiographic analysis of cardiac function (EF, ejection fraction; FS, fractional shortening; mean ± s.e.m.; n = 6 mice per group). The mice were 8–12 weeks old.
(h) Electron micrographs of left ventricular tissue (scale bars, 1 μm).
(i, j) Individual mitochondrial area and total volume fraction quantified from electron micrographs as shown in **(h)** (mean ± s.e.m.; n = 73–139 mitochondria for **(i)** and 9–17 images for **(j).**
(k) ATP content of WT and TG cardiomyocytes (mean ± s.e.m.; n = 5–6 mice per group). The cardiomyocytes were isolated form 8–12 weeks old mice.
(l) Mitochondrial DNA content in WT and TG mouse heart (mean ± s.e.m.; n = 5-6 mice).
