# Supplementary material for: NDUFAB1 confers cardio-protection by enhancing mitochondrial bioenergetics through coordination of respiratory complex and supercomplex assembly
Source: Cell Res. 2019 Jul 31;29(9):754–66. doi: 10.1038/s41422-019-0208-x (PMC6796901; doi:10.1038/s41422-019-0208-x)
Supplement: Supplementary file 14 — Supplementary information Fig. S14 [file 41422_2019_208_MOESM14_ESM.pdf]

Fig. S14

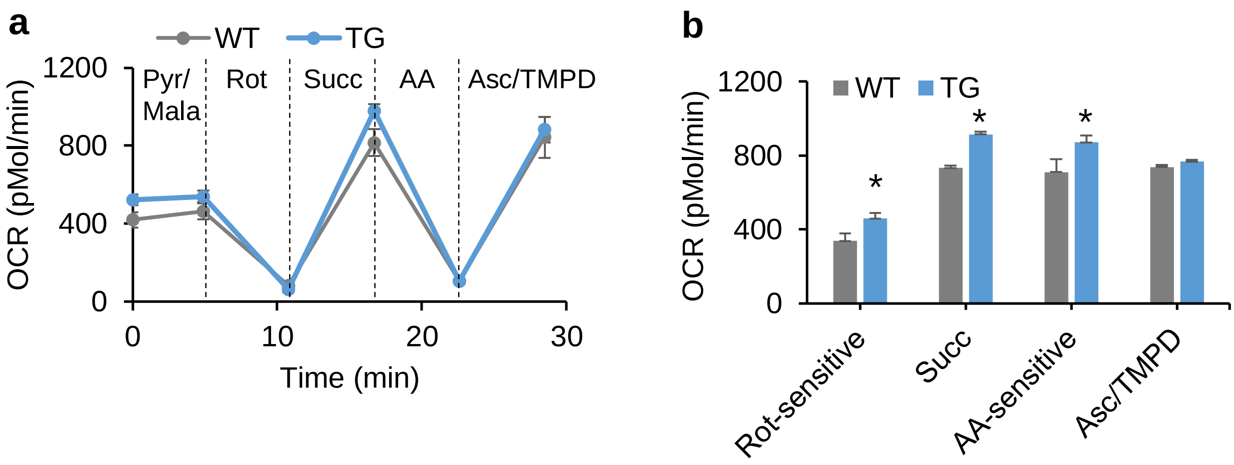

Fig. S14. Electron flow assay of isolated cardiac mitochondria from the WT and TG mice.

(a) Mitochondrial OCR measured with Seahorse under different conditions. Dashed lines indicate the sequential additions of 10 mM pyruvate, 2 mM malate and and 4 μM FCCP (Pyr/Mala), 2 μM rotenone (Rot), 10 mM succinate (Succ), 4 μM antimycin A (AA), and 10 mM ascorbate and 100 μM TMPD (Asc/TMPD).

(b) Statistics of the activities of complexes I-IV in (**a**). Data are mean ± s.e.m.; * $p<0.05$ versus WT. n = 10-17 wells from 4 mice per group.
