# Supplementary material for: NDUFAB1 confers cardio-protection by enhancing mitochondrial bioenergetics through coordination of respiratory complex and supercomplex assembly
Source: Cell Res. 2019 Jul 31;29(9):754–66. doi: 10.1038/s41422-019-0208-x (PMC6796901; doi:10.1038/s41422-019-0208-x)
Supplement: Supplementary file 15 — Supplementary information Fig. S15 [file 41422_2019_208_MOESM15_ESM.pdf]

Fig. S15

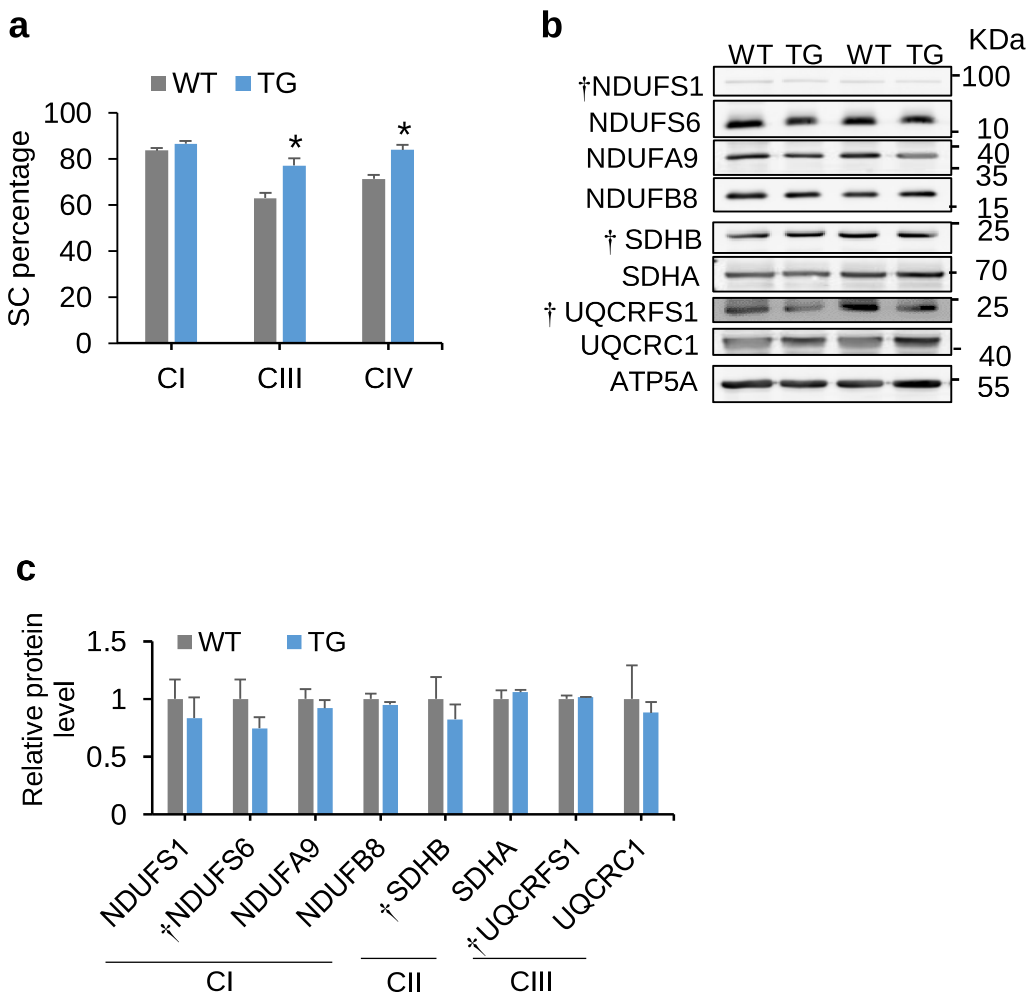

Fig. S15. Changes of supercomplex percentages and individual ETC complex subunits in TG hearts.

(a) The percentages of SCs in TG mitochondria (mean ± s.e.m.; n = 4-8 mice per group, *p <0.05 versus WT).

(b) Western blots for subunits of complexes I-III in *Ndufab1* TG and WT cardiomyocytes. ATP5A served as the loading control. †FeS-containing subunits.

(c) Statistics of **b** (mean ± s.e.m.; n = 3 male mice per group).
