# Supplementary material for: NDUFAB1 confers cardio-protection by enhancing mitochondrial bioenergetics through coordination of respiratory complex and supercomplex assembly
Source: Cell Res. 2019 Jul 31;29(9):754–66. doi: 10.1038/s41422-019-0208-x (PMC6796901; doi:10.1038/s41422-019-0208-x)
Supplement: Supplementary file 16 — Supplementary information Fig. S16 [file 41422_2019_208_MOESM16_ESM.pdf]

Fig. S16

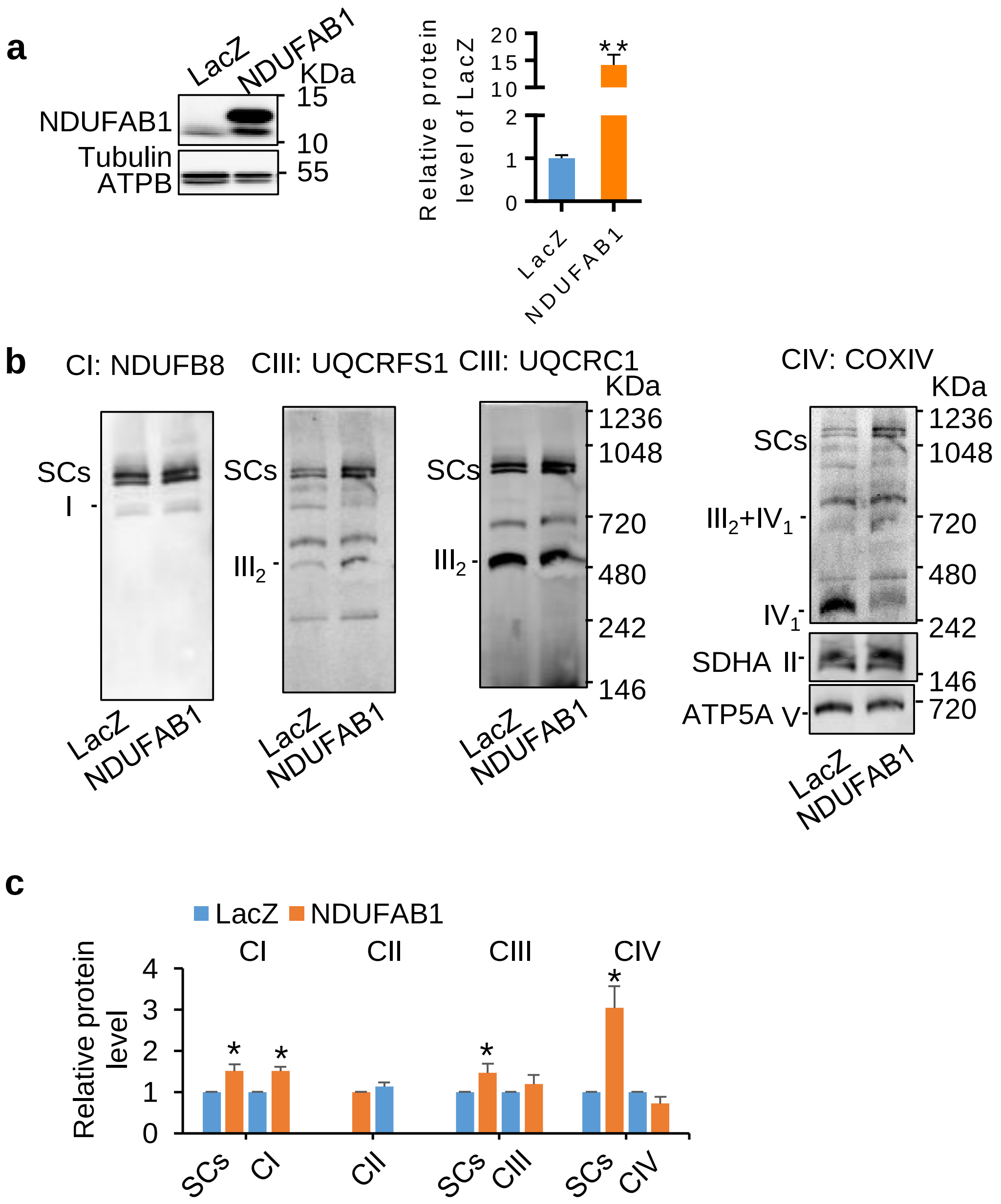

Fig. S16. Effects of *Ndufab1* overexpression on the assembly of ETC complexes and SCs in cultured neonatal rat cardiomyocytes.

(a) Western blots of NDUFAB1 in *Ndufab1* overexpression cardiomyocytes. ATPB served as the loading control. NDUFAB1: *Ndufab1* overexpression adenovirus; LacZ: LacZ adenovirus as the control. Data are mean ± s.e.m.; n = 4 per group; ** p<0.01 versus LacZ.

(b) BN-PAGE immunoblots of individual ETC complexes and SCs. The antibodies used were as the same as Figure 3c.

(c) Statistics of **(b)**. The expression was normalized to LacZ group (mean ± s.e.m.; n = 3–4 per group; * p<0.05 versus LacZ). For CIII, anti-UQCRFS1 blots were used.
