# Supplementary material for: NDUFAB1 confers cardio-protection by enhancing mitochondrial bioenergetics through coordination of respiratory complex and supercomplex assembly
Source: Cell Res. 2019 Jul 31;29(9):754–66. doi: 10.1038/s41422-019-0208-x (PMC6796901; doi:10.1038/s41422-019-0208-x)
Supplement: Supplementary file 18 — Supplementary information Fig. S18 [file 41422_2019_208_MOESM18_ESM.pdf]

Fig. S18

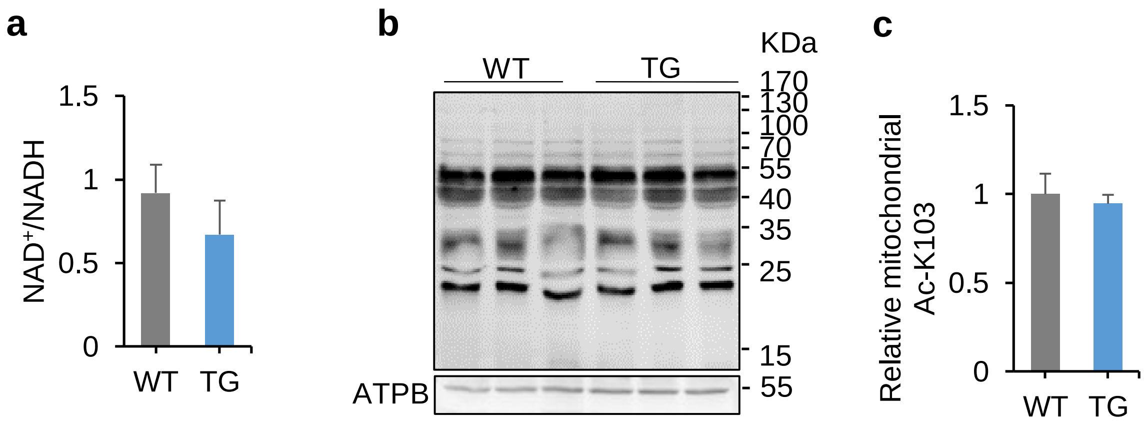

Fig. S18. NAD$^+$/NADH ratio and protein acetylation in TG and WT hearts.
(a) NAD$^+$/NADH ratio in the TG and WT hearts (mean ± s.e.m.; n = 3 mice per group).
(b) Representative western blots of the acetylome in the TG and WT hearts. ATPB served as the loading control.
(c) Statistics of **(b)** (mean ± s.e.m.; n = 3 mice per group).
