# Supplementary material for: NDUFAB1 confers cardio-protection by enhancing mitochondrial bioenergetics through coordination of respiratory complex and supercomplex assembly
Source: Cell Res. 2019 Jul 31;29(9):754–66. doi: 10.1038/s41422-019-0208-x (PMC6796901; doi:10.1038/s41422-019-0208-x)
Supplement: Supplementary file 19 — Supplementary information Fig. S19 [file 41422_2019_208_MOESM19_ESM.pdf]

Fig. S19

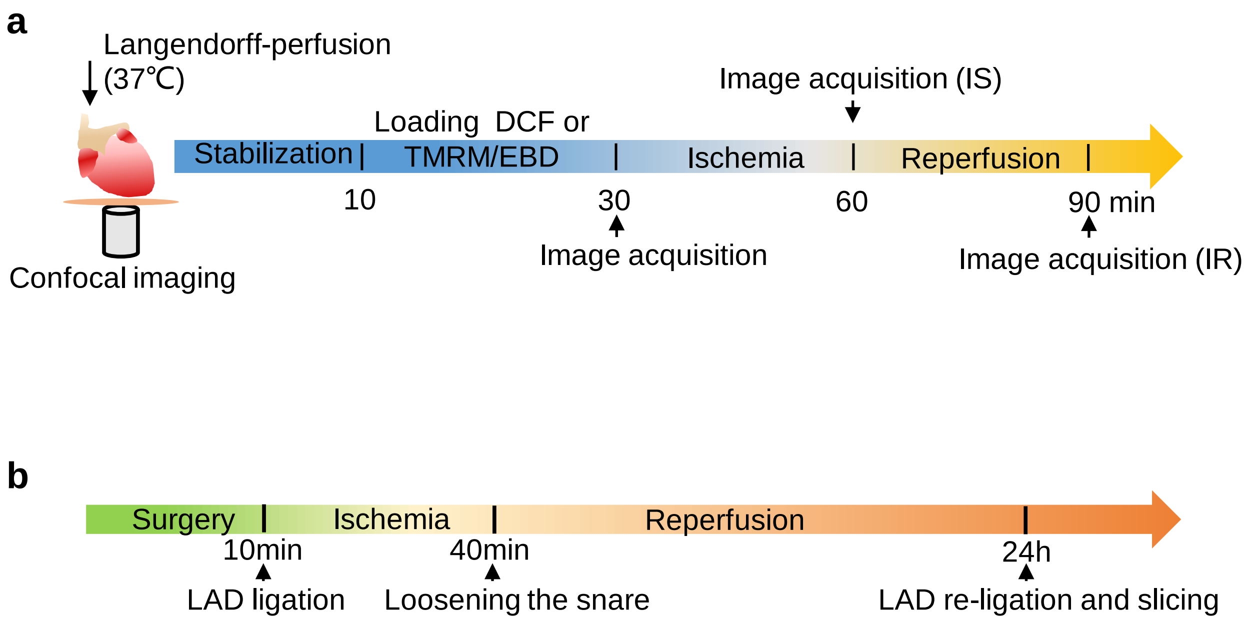

Fig. S19. Schematic of *ex vivo* and *in vivo* IR protocols.

(a) Schematic of the *ex vivo* IR protocol for the Langendorff-perfused heart. Briefly, the heart was subjected to ischemia for 30 min and then reperfusion for 30 min. Images were captured before ischemia, at 30 min of ischemia (IS), and at 30 min of reperfusion (IR).

(b) Schematic of the *in vivo* IR protocol. A reversible coronary artery snare occluder was placed around the left anterior descending (LAD) coronary artery of the anesthetized mouse. Myocardial I/R was induced by tightening the snare for 30 min and then loosening it for 24 h.
